# Heterogeneous Patterns of Genetic Diversity and Differentiation in European and Siberian Chiffchaff (*Phylloscopus collybita abietinus/P. tristis*)

**DOI:** 10.1534/g3.117.300152

**Published:** 2017-10-20

**Authors:** Venkat Talla, Faheema Kalsoom, Daria Shipilina, Irina Marova, Niclas Backström

**Affiliations:** * Department of Evolutionary Biology, Evolutionary Biology Centre, Uppsala University, 752 36, Sweden; † Department of Vertebrate Zoology, Lomonosov Moscow State University, 119991, Russia

**Keywords:** chiffchaff, speciation, genome-scan, divergence islands, Z-chromosome, autosomes

## Abstract

Identification of candidate genes for trait variation in diverging lineages and characterization of mechanistic underpinnings of genome differentiation are key steps toward understanding the processes underlying the formation of new species. Hybrid zones provide a valuable resource for such investigations, since they allow us to study how genomes evolve as species exchange genetic material and to associate particular genetic regions with phenotypic traits of interest. Here, we use whole-genome resequencing of both allopatric and hybridizing populations of the European (*Phylloscopus collybita abietinus*) and the Siberian chiffchaff (*P. tristis*)—two recently diverged species which differ in morphology, plumage, song, habitat, and migration—to quantify the regional variation in genome-wide genetic diversity and differentiation, and to identify candidate regions for trait variation. We find that the levels of diversity, differentiation, and divergence are highly heterogeneous, with significantly reduced global differentiation, and more pronounced differentiation peaks in sympatry than in allopatry. This pattern is consistent with regional differences in effective population size and recurrent background selection or selective sweeps reducing the genetic diversity in specific regions prior to lineage divergence, but the data also suggest that postdivergence selection has resulted in increased differentiation and fixed differences in specific regions. We find that hybridization and backcrossing is common in sympatry, and that phenotype is a poor predictor of the genomic composition of sympatric birds. The combination of a differentiation scan approach with identification of fixed differences pinpoint a handful of candidate regions that might be important for trait variation between the two species.

Characterization of the genetic basis of reproductive isolation is essential for understanding the speciation process, and identification of candidate genes that may underlie trait variation related to reproductive barriers is a key step in this process (Feder *et al.* 2017; Westram and Ravinet 2017). Until recently, speciation genetic research revolved predominantly around identification of genes that lie behind intrinsic postzygotic incompatibilities between deeply divergent lineages in the laboratory (Wolf *et al.* 2010; Coyne and Orr 2004; Noor and Feder 2006). However, recent developments in DNA sequencing technology and genome analysis have paved the way for detailed investigations of the genetic underpinnings of reproductive isolation also in species that previously have lacked genomic tools (Baird 2017). These technological advancements hold the promise of contributing to the discovery of genetic components underlying traits that contribute to species divergence in natural settings, both at early and late stages of the divergence process (Storz 2005; Wolf and Ellegren 2017; Seehausen *et al.* 2014). When lineages diverge, they may evolve allele combinations that are coadapted and lineage specific (Wu and Ting 2004). As a consequence, genetic incompatibilities may arise in hybrids (Bateson 1909; Dobzhansky 1940; Muller 1942) when divergent lineages meet and interbreed (Coyne and Orr 2004; Turelli and Orr 2000). The effect may be seen in first generation hybrids, but should be particularly pronounced in later stage backcrosses (*e.g.*, Qvarnström *et al.* 2010), and can lead to reinforcement of the speciation progression via selection against interspecific pairings and backcrosses (*e.g.*, Sætre *et al.* 1997; Sætre and Sæther 2010). Hybrid zones, regions where distribution ranges of nascent species coincide and interspecific crosses occur, therefore provide an ultimate study system for identification of genetic elements underlying initialization and establishment of barriers to gene flow in natural settings (Jiggins *et al.* 1996; Barton and Hewitt 1989; Abbott *et al.* 2016; Payseur and Rieseberg 2016).

Of late, there has been gradually increasing interest in understanding how adaptation to the local environment can drive the formation of reproductive barriers between diverging populations (Nosil and Feder 2012). This has been termed “ecological speciation,” and refers to a scenario where reproductive isolation evolves as a side-effect of adaptation to the environment (Schluter 2009). Indirect support for ecological speciation predominantly comes from experimental evolution investigations in captive populations of micro-organisms (*e.g.*, Buckling *et al.* 2009), but also from a handful of studies in free-ranging natural populations (*e.g.*, Via 2009; Bernatchez *et al.* 2010; Chung *et al.* 2014; Gompert *et al.* 2014; Egan *et al.* 2015). At early stages of population divergence, it is expected that genetic loci governing traits that affect adaptation will be more differentiated than the genomic average (Nosil and Feder 2012; Seehausen *et al.* 2014; Wolf and Ellegren 2017). Theoretically, this allows for using genome scans for diversifying selection in incipient species or differentiated populations to identify genes that might be, or have been, involved in the speciation process (Wolf *et al.* 2010; Seehausen *et al.* 2014; Wolf and Ellegren 2017). Importantly, selection may also reduce the interspecific recombination rate in regions that are in physical linkage with the selected locus (Via and West 2008). The size of such regions may vary, and depends primarily on the relative strength of selection and the local rate of recombination (Charlesworth *et al.* 1997); in some cases, large genomic regions of chromosomes can show this pattern (Via and West 2008; Feder and Nosil 2010; Rogers and Bernatchez 2007); in others, a very restricted portion or a single gene may underlie both ecological divergence and reproductive isolation (Chung *et al.* 2014; Joron *et al.* 2006; Martin *et al.* 2013). Hence, by applying a combination of high-throughput DNA sequencing techniques with population genomic methods to identify loci that deviate from the expectations from neutrality, it is now a realistic goal to perform genome-scans for genes involved in adaptation, and to try to identify a potential link to speciation also in nonmodel species (Storz 2005).

Recent genome-scan approaches using population samples from a range of taxa at different levels of divergence have shown that a common pattern encountered is a mosaic of highly and lowly differentiated genomic regions (Ellegren *et al.* 2012; Turner *et al.* 2005; Martin *et al.* 2013; Renaut *et al.* 2013; Ferchaud and Hansen 2016; Jones *et al.* 2012; Wolf and Ellegren 2017; Burri *et al.* 2015; Vijay *et al.* 2016; Harr 2006; Poelstra *et al.* 2014). This is in accordance with a scenario where gene-flow has reduced the level of genetic differentiation in general, but where particular genomic regions are sheltered from intermixing of parental alleles, and highly differentiated regions (so called “speciation islands” or “differentiation islands”) were therefore initially interpreted to contain incompatibility loci. However, with the recognition that a heterogeneous differentiation landscape may evolve also without gene-flow (Cruickshank and Hahn 2014), elevated differentiation may for example reflect low ancestral genetic diversity, and/or be a side-effect of reduced regional effective population size as a consequence of lower than average recombination rate (Burri *et al.* 2015; Noor and Bennett 2009), the interpretations of patterns of genomic differentiation have become more careful (Pennisi 2014; Ravinet *et al.* 2017). Although natural selection is a key driver toward patterns of heterogeneous differentiation between diverging lineages with or without gene-flow (Cruickshank and Hahn 2014), linking patterns of genomic differentiation to reproductive isolation is challenging (Ravinet *et al.* 2017). One way forward is to have robust and well-planned study designs with genome-wide data of populations at different stages of divergence, and/or including data from multiple geographical locations involving both allopatric and sympatric regions, including potential hybrid zones (Seehausen *et al.* 2014). Detailed analysis of population genetic summary statistics in allopatry and in sympatry complemented by comparisons between empirical and theoretical distributions to detect significant outliers among statistics can assist in the understanding of the initiation and establishment of divergence process underlying observed patterns of differentiation, and can shed light on the progression of the speciation process (Seehausen *et al.* 2014; Wolf and Ellegren 2017), with or without gene-flow (Feder *et al.* 2013, 2012).

Here, we present a population genomic survey using both allopatric and sympatric samples of the common chiffchaff (*Phylloscopus collybita abietinus*) and Siberian chiffchaff (*P. tristis*) as a study system to quantify patterns of genetic diversity and differentiation across the genome. The chiffchaff superspecies complex is part of the Eurasian Old World leaf warbler group, and, historically, the complex has been divided in four distinct species: Canarian chiffchaff, Iberian chiffchaff, mountain chiffchaff, and common chiffchaff (Helbig *et al.* 1996). The common chiffchaff (*P. collybita*) has been divided into five different subspecies, including subspecies *abietinus* (eastern European common chiffchaff) and *tristis* (Siberian chiffchaff). *P. tristis* has recently been given full species status (del Hoyo and Collar 2017), now being considered a sister species to *P. collybita*, and we therefore refer to common chiffchaff and Siberian chiffchaff as distinct species. Since our particular focus concerns the comparison of *P. tristis* to one of the European common chiffchaff subspecies (*P. c. abietinus*) only, and to accommodate consistency with recent previous analyses of this particular pair of taxa (Shipilina *et al.* 2017), we will refer to the two species as *abietinus* and *tristis* from here on. The breeding range of *abietinus* covers the regions from north-eastern Europe to the Ural mountains, and the seasonal migration goes to wintering areas in eastern and central Africa (del Hoyo *et al.* 2006; Stepanyan 1990). The Siberian chiffchaff (*tristis*) is distributed from the Ural mountains to eastern Siberia during breeding season, and migrates to south-central and south-eastern Asia (del Hoyo *et al.* 2006). Previous analyses using both phenotypic, acoustic, and genetic data have shown that *abietinus* and *tristis* differ significantly in appearance, body size, vocalizations, habitat preference, and migration patterns (Komarova and Shipilina 2010; Marova and Alekseev 2008; Marova *et al.* 2009, 2013, 2017; Marova and Leonovich 1993; Martens and Meinche 1989; Ticehurst 1938; Van den Berg 2009; Svensson 1992), and that they co-occur in a zone of secondary contact in a region from the southern Ural mountains in the south to the White Sea in the north, where they commonly hybridize and back-cross (Marova *et al.* 2017; Shipilina *et al.* 2017). *Abietinus* is more yellowish-green in plumage color, has a slightly larger body size, sings a slower and more rhythmic song with wider frequency range, and prefers mixed forests, whereas *tristis* has a duller appearance with no yellow tones (except on under wing coverts), is smaller in size, sings a faster and more diverse song, and preferably inhabits boreal forests (Marova and Alekseev 2008; Marova *et al.* 2009, 2013, 2017). In the sympatric zone, distinct *abietinus* and *tristis* individuals appear, but a relatively large proportion of individuals are characterized by showing intermediate plumage color, singing a mixed song type, and having a genetic composition consisting of a mixture of diagnostic *abietinus* and *tristis* alleles (Marova *et al.* 2009; Shipilina *et al.* 2017; Lindholm 2008; Ticehurst 1938). This makes the *abietinus* and *tristis* species pair an ideal model system to characterize the patterns of genomic differentiation as a result of divergence in allopatry followed by recurrent hybridization and back-crossing in sympatry. Our aims with the study were to: i) use population whole genome resequencing data to get global (genome-wide) and regional (locus specific) estimates of diversity and differentiation in allopatry and sympatry, ii) to use a genome-scan approach to characterize the landscape of differentiation between typical *abietinus* and *tristis* morphotypes in allopatry and in sympatry to detect regions that appear impermeable to gene-flow, and iii) to identify candidate genes for local adaptation and reproductive isolation between the species.

## Materials and Methods

### Sampling information and DNA sequencing

Male individuals of both *abietinus* and *tristis* were collected in the field during breeding seasons 2007–2009 (for details, see Shipilina *et al.* 2017). Ten allopatric and 10 sympatric birds were selected for population resequencing from each of the two species—in total 40 birds. All birds were captured using mist-netting with song traps, and blood samples were collected using a standard syringe to puncture the brachial vein. Blood was stored on filter paper and DNA was extracted using a standard phenol-chloroform protocol (Sambrook *et al.* 1989). Illumina, paired-end, individually barcoded, libraries with 380-bp insert sizes were constructed, and sequenced in a multiplex set-up in two separate batches on an Illumina HiSeq2000 sequencer—in total, all 40 samples were run in multiplex on eight lanes, generating, on average, 3.7 Gb of raw sequence data for each individual (Supplemental Material, Table S1 in File S1). A single allopatric *abietinus* male (*Pcol09*) was selected for deep sequencing, and two different paired-end libraries (180 and 380 bp) were generated for this individual and sequenced on four lanes—this generated, in total, 100.2 Gb of raw sequence data (91.1 Gb after filtering out bases with Q-scores <30) for this individual (Table S1 in File S1). Libraries were prepared by the SNPandSEQ Technology Platform in Uppsala, and sequencing was performed in two separate batches, one batch using Illumina HiSeq2000 technology (100 bp read length) at the SNPandSEQ Technology Platform in Uppsala (four lanes with 10 samples per lane, data also used in Shipilina *et al.* 2017), and a supplementary batch using the same technology at the National Genomics Infrastructure (NGI) center in Stockholm (again four lanes with 10 samples per lane). All sequence reads have been deposited in the European Nucleotide Archive (http://www.ebi.ac.uk/ena) under accession number: PRJEB21643 (Table S1 in File S1). The sample names *Pcol01-Pcol704* follow the nomenclature established during the sampling stage, when *abietinus* and *tristis* were classified as subspecies of the common chiffchaff (*P. collybita*; *i.e.*, *P. c. abietinus and P. c. tristis*), and we decided to keep the sample names for easier comparison of results from studies of the same system (Shipilina *et al.* 2017).

### Assembly and quality assessment

A reference-assisted nuclear genome assembly of the *Pcol09* individual was generated using version fAlb15 of the collared flycatcher (*Ficedula albicollis*) assembly as a reference (Ellegren *et al.* 2012; Kawakami *et al.* 2014). First, paired-end sequence reads from *Pcol09* (>100× sequence coverage in total for the two different insert size libraries) were aligned to the *F. albicollis* assembly using BWA version 0.7.8 (Li and Durbin 2010). The consensus sequence from all mapped reads with mapping quality ≥30, and a coverage between 5× and 50× per base was extracted from the resulting BAM file using the mpileup function in SAMtools version 1.3 (Li *et al.* 2009). The vcfutils function in BCF-tools was used to convert the assembly VCF-file into a FASTQ-file, and seqtk (https://github.com/lh3/seqtk) was used for the conversion of the FASTQ-file format to a FASTA formatted final assembly. Ambiguous bases in the reference-assisted assembly were replaced by N if they represented more than two alleles, and diallelic ambiguities were assigned to either allele randomly using an inhouse developed python script (https://github.com/venta380/chiffchaff_project/). The reference assisted chiffchaff genome assembly covered 89% of the entire *F. albicollis* reference genome, and spanned, in total, 1.04 Gb. It should be noted that a reference assisted assembly is blind to structural changes and differences in synteny can affect the interpretation of genome-wide patterns of genetic variation. Although the divergence time of *Ficedula* and *Phylloscopus* is ∼40 MY (Claramunt and Cracraft 2015; Selvatti *et al.* 2015), the avian karyotype is remarkably conserved (Ellegren 2009), and the reference-assisted chiffchaff assembly likely contains only a limited number of structural inconsistencies, with a minor effect on the main conclusions of this study. The quality of the chiffchaff assembly was evaluated using the QUAST (Quality Assessment Tool for Genome Assemblies) software version 3.2 (Gurevich *et al.* 2013), and by browsing for presence of conserved vertebrate and eukaryote gene sets using BUSCO (Benchmarking Universal Single-Copy Orthologs) version 1.1b1 (Simao *et al.* 2015). The quality assessment results obtained for the chiffchaff assembly were compared to BUSCO results for the *F. albicollis* reference assembly and for the crow (*Corvus corone*) genome assembly, to obtain comparative data for high-quality avian genome assemblies. In order to assure sufficient mapping efficiency of the population resequencing data, one random sample (*Pcol41*) from the resequenced sample set was mapped to the chiffchaff assembly using BWA version 0.7.8 (Li and Durbin 2010), and mapping coverage and depth were computed using SAM-tools version 1.3 (Li *et al.* 2009) and BED-tools version 2.25.0 (Quinlan and Hall 2010). All assembly and quality assessment statistics and comparisons to reference bird species (*F. albicollis* and *C. corone*) are presented in Table S2 and Table S3 in File S1.

Complete chiffchaff mtDNA genome assemblies were generated for each of the 40 samples using mapping with BWA (Li and Durbin 2010), with the previously available *F. albicollis* mitochondrial genome as template to identify mtDNA reads. Mapped reads were subsequently used in *de novo* assemblies of chiffchaff mtDNA genome assemblies using the iterative mapping and assembly tool MITObim (Hahn 2013). The procedure was iterated five times using the most recently generated chiffchaff instead of the *F. albicollis* mtDNA assembly as a template. The final chiffchaff mtDNA assemblies were ∼17,000 bp long, and we annotated them with the web server MITOS (Bernt *et al.* 2013). A comparison between the *F. albicollis* and the chiffchaff mtDNA assembly for individual *Pcol09* (the individual with deepest sequencing coverage also used for reference assisted genome assembly) revealed that gene order and gene sizes were extremely conserved between the two lineages (Figure S1 in File S1)—note that the mtDNA genome is circular and the presentation in linear format generates a random cut point so that start and stop positions are different in the two assemblies.

### Population resequencing and variant calling

The chiffchaff assembly was prepared to be used as a reference for mapping by indexing to different formats compatible with the GATK (Genome Analysis Tool Kit) SNP calling algorithms using BWA version 0.7.8 (Li and Durbin 2010) and SAMtools version 1.3 (Li *et al.* 2009). An assembly sequence database with scaffold ID, scaffold size, and genomic anchoring positions on the *F. albicollis* reference genome was created using Picard Tools version 1.127 (available at: https://github.com/broadinstitute/picard). All 40 resequenced samples were mapped individually to the chiffchaff reference assembly using BWA version 0.7.8 (Li and Durbin 2010). The resulting SAM (Sequence Alignment/Map) files were converted into BAM (Binary Alignment/Map) files and merging of BAM-files for different sequence sets for each respective individual was done using SAMtools version 1.3 (Li *et al.* 2009). In order to prepare the obtained 40 BAM files for the variant calling step, different preprocessing steps of the GATK (Genome Analysis Tool Kit) best practices were performed (McKenna *et al.* 2010); the PCR duplicates were marked and deleted with MarkDuplicates option in Picard tools version 1.127 (available at: https://github.com/broadinstitute/picard), and the sequences flanking indels were realigned using IndelRealigner in GATK (McKenna *et al.* 2010). Then, all reads from the individual used for reference assisted genome assembly (*Pcol09*) were mapped back to the assembly using BWA (Li and Durbin 2010), and the HaplotypeCaller in GATK (McKenna *et al.* 2010) was run to generate an initial set of variants. The resulting VCF (Variant Call Format) file obtained from this analysis was used together with the BaseRecalibrator utility in GATK (McKenna *et al.* 2010) to call a reference set of variants for the recalibration of bases in all the population samples. The quality of the recalibrated BAM files was checked with QualiMap version 2.0.2 (Okonechnikov *et al.* 2015). One sample (*Pcol703*, an allopatric *tristis* individual) was found to have considerably lower mapping quality than all other samples, and was removed from the downstream analysis in order to reduce biases in the results as a consequence of differences in mapping efficiency, coverage, and variant calling. Sequence polymorphisms were called for each sample individually using HaplotypeCaller, subsequently joint genotyped using GenotypeGVCFs, and filtered using standard settings for the VariantRecalibrator in GATK (McKenna *et al.* 2010).

### Assignment analysis and visualization of genetic relationships

#### Population structure:

A SNP-based principal component analysis (PCA) was conducted on genotypes that were covered with a minimum of seven reads per individual in all samples in each of the four populations. The PCA was performed with 220,097 SNPs using the R Bioconductor package SNPRelate (Zheng 2012), using a linkage disequilibrium threshold of *r^2^* < 0.2 (https://github.com/venta380/chiffchaff_project/). A population structure analysis was conducted using the R Bioconductor package LEA (Landscape and Ecological Association Studies) (Frichot 2015) on the same set of 220,097 high-quality SNPs used for the PCA analysis. The LEA package uses a program called sNMF (Frichot 2014) to cluster multi-locus genotypes based on a set value of number of populations (K) provided to the program. The range of K-values were set to be from 1 to 8 to find out the optimal number of populations explaining the data using cross-entropy values (Frichot 2014, 2015).

#### Mitochondrial DNA:

The mitochondrial genome was assembled for each individual independently (see above), and all mtDNA genome sequences were aligned with MAFFT version 7 (Katoh and Standley 2013), generating an alignment of 17,240 bp. The alignment was visually inspected for potential errors and ends (representing parts of the hypervariable D-loop region) were discarded to avoid false signals in the analysis of relationships. In total, 16,998 bp were used for the subsequent analyses. A branch length scaled mtDNA genome phylogeny was generated using Neighbor-Joining (Saitou and Nei 1987). The distances (substitutions per site) were computed using the Maximum Composite Likelihood (Tamura *et al.* 2004), and rate variation among sites was modeled using a gamma distribution with shape parameter 1 as implemented in MEGA7 (Kumar *et al.* 2016). Ambiguous positions were removed for pairwise comparisons. The mtDNA genome alignment was used to infer intrapopulation levels of nucleotide diversity by averaging over all sequence pairs in each respective population, and omitting missing data in pairwise comparisons using the Maximum Composite Likelihood method (Tamura *et al.* 2004) as implemented in MEGA7 (Kumar *et al.* 2016). We also estimated average mtDNA genome sequence differentiation across population pairs, again averaging over cross-population sequence pairs using the Maximum Composite Likelihood method (Tamura *et al.* 2004) as implemented in MEGA7 (Kumar *et al.* 2016), and ignoring missing data in pairwise comparisons.

### Genome resequencing analysis

#### Repeat filtering:

Before any additional analyses, interspersed and tandem repeats in the chiffchaff assembly were hard masked using RepeatMasker version 4.0.6 (Smit *et al.* 2013–2015), using previously available *F. albicollis* repeat annotation libraries (https://www.ncbi.nlm.nih.gov/genome/annotation_euk/Ficedula_albicollis/101/) as reference.

#### Genotype filtering:

The sequence coverage depth at each genotype was calculated using the geno-depth option in Vcf-tools version 0.1.14 (Danecek *et al.* 2011). To avoid considerable biases in allele frequency estimates in downstream analysis, only SNPs that were present in at least seven individuals in each respective population [allopatric *abietinus* (*n* = 10), allopatric *tristis* (*n* = 9), sympatric *abietinus* (*n* = 10), and sympatric *tristis* (*n* = 10)], with a minimum sequence coverage of five reads (*i.e.*, each base had to be covered by at least five different reads), were retained for subsequent analysis. Genotype calls were translated to specific polymorphism classes for both the allopatric and sympatric species comparison; fixed differences between the two species, private polymorphisms in *abietinus*, private polymorphisms in *tristis*, and shared SNPs (polymorphic in both species).

#### Population genetic summary statistics:

The ANGSD (Analysis of Next Generation Sequencing Data) package version 0.902 (Korneliussen *et al.* 2014) was used to calculate population genetic summary statistics within each of the populations [pair-wise nucleotide diversity (*Ɵ_π_*), Watterson’s theta (*Ɵ_W_*) and Tajima’s *D*] and genetic differentiation (*F_ST_*) between allopatric and sympatric species pairs. As the ancestral state for segregating sites was not known, the folded allele frequency likelihoods and allele frequency spectra were computed using GATK’s genotype likelihood model as implemented in ANGSD (Korneliussen *et al.* 2014). We used inhouse developed python scripts (https://github.com/venta380/chiffchaff_project/) to calculate absolute divergence (*D_XY_*) between allopatric and sympatric species pairs, and independent estimates of *Ɵ_π_* for each respective population based on the allele frequencies estimated using ANGSD. The rationale behind this was that the inhouse calculated *D_XY_* estimates, and the *Ɵ_π_* estimates from ANGSD were not calculated using identical settings, and to compare intraspecific polymorphism levels with absolute divergence we needed to apply the same settings for these summary statistics. All summary statistics were averaged over nonoverlapping windows of 10 kb across the genome. The levels of linkage disequilibrium (LD, estimated with the correlation between alleles at different loci: *r^2^*) for each respective population were calculated for SNP pairs <100 kb apart using Plink version 1.90 (Purcell *et al.* 2007). To avoid inflated LD estimates due to low allele frequencies, we only included SNPs with minor allele frequency (MAF) > 0.2. The population specific average LD per SNP pair in each 10-kb window was calculated using custom python scripts (https://github.com/venta380/chiffchaff_project/), with the filtered LD output from each population specific analysis from Plink (Purcell *et al.* 2007).

To assess the genomic distribution of the most highly differentiated regions between *abietinus* and *tristis*, and identify differentiation outliers, we Z-transformed the *F_ST_* estimates (*F_ST_^Z^*) for each comparison by standardizing with the genomic average and variance across windows according to the following formula:

$$F_{ST}^{Z}=\frac{F_{ST}\;\text{for}\;\text{window}-\text{mean}\;\text{genomewide}\;F_{ST}}{\text{SD}\;\text{of}\;\text{genomewide}\;F_{ST}}$$

The estimates were calculated for the autosomes and the Z-chromosome separately since the effective population size (*N_e_*) for the Z-chromosome was reduced as compared to the autosomes, and, hence, the expected baseline levels differ between these chromosome classes. Autosomal windows with *F_ST_^Z^* values higher than the 99th percentile in the distributions were considered outliers (≥4 SD higher than the global mean) for the allopatric and the sympatric comparison, respectively. In order to evaluate if the overall sequence coverage affected the *F_ST_* distribution (hypothetically the level of differentiation may be biased upwards as a result of unusual low coverage), per-site genome coverage was calculated using the coverageBed utility in the BEDTools version 2.25.0 (Quinlan and Hall 2010), and the correlation between the coverage and *F_ST_* was checked. We found only weak and inconsistent correlations between sequence coverage and *F_ST_* (range of Pearson’s *r* = −0.007 to 0.025 for the four different populations). To visually inspect the landscape of diversity and differentiation in the allopatric and the sympatric species comparisons, all population genetic summary statistics were plotted against physical positions along chromosomes using the ggplot2 library in R (https://cran.r-project.org/). The most extreme differentiation regions in each comparison (*F_ST_^Z^* > 99th percentile in the empirical distribution) were further investigated by extracting the coordinates for clusters (≥10 outlier windows present in a 0.5-Mb genomic region) of the windows with the highest level of relative differentiation, and intersect these with annotation data from the *F. albicollis* genome (http://www.ensembl.org/Ficedula_albicollis/Info/Index: 2017-06-30). The rationale behind this strategy was to identify coding genes to investigate if any particular functional category of genes was over-represented in the most highly differentiated regions. We used a binomial distribution to estimate the probability of this level of clustering or higher, since the sample size (50 windows per block) was much smaller than the total population (103,800 windows in total; 1038 outlier windows). The probability of 10 outlier windows clustering in a 500 kb (50 windows) region was estimated at 7.133 × 10^−11^. Importantly, relaxing the significance level did not affect the results quantitatively, *i.e.*, very few additional blocks were identified if the limits were set to 5 (p-value ≈ 0.0001) or even three outliers (p-value ≈ 0.01) within a 50-window block (data not shown). Enrichment analysis of specific gene ontology (GO) terms for genes located in chromosome regions with higher than expected density of relative differentiation outliers was performed using GOstat with a custom reference database based on GO terms from the *F. albicollis* gene set as available in Ensembl (http://www.ensembl.org/Ficedula_albicollis/Info/Index: 2017-06-30). The identities and potential functions of the identified genes in highly differentiated regions were inferred using orthology information as available in the Biomart tool in Ensembl (http://www.ensembl.org/biomart/martview/c02baa1e6506b4840e7e34ceac0a49da: 2017-06-30).

### Functional analysis of fixed, shared, and private polymorphisms

To further assess the potential functional relevance of different types of polymorphisms, we used custom PyVCF 0.6.8 scripts (https://github.com/venta380/chiffchaff_project/) to sort previously called SNPs into four categories for the allopatric species comparison (as mentioned above, we found no fixed differences between sympatric *abietinus* and *tristis*): fixed differences between the two species, private polymorphisms in *abietinus*, private polymorphisms in *tristis* and shared SNPs (polymorphic in both species). Only SNPs covered by at least five reads in each individual and where information was present for at least nine individuals in each of the populations were retained for this analysis (*n* = 2,810,678) to minimize the bias toward fixed differences when representation in individuals is low. We identified the functional relevance of each SNP using the annotation software snpEFF version 4.3 (Cingolani *et al.* 2012) with a manually built custom database based on genome annotation information from *F. albicollis* (http://www.ensembl.org/Ficedula_albicollis/Info/Index), which is the closest relative to chiffchaff with extensive genome annotation information. The annotations were grouped into classes “downstream region,” “upstream region,” “exon,” “intron,” “intergenic region,” and “other” (the latter including 3′UTR, 5′UTR, and splice sites). The relative frequencies of different polymorphisms were then compared across annotation classes for autosomes and the Z-chromosome separately to get information about the relative effects of selection and genetic drift in driving the patterns of differentiation, heterozygosity, allele sharing, and accumulation of fixed differences between *abietinus* and *tristis*.

### Data availability

All sequence reads in this study have been deposited in the European Nucleotide Archive under project accession number PRJEB21643. Individual accession numbers for all individuals are available in Table S1 in File S1.

## Results

### Assembly statistics

The total length of the chiffchaff assembly was 1.04 Gb, similar to previously available avian genome assemblies (Zhang *et al.* 2014). The longest scaffold was 157.6 Mb, and the scaffold N50 length was 70.4 Mb, identical to the fAlb15 assembly (Kawakami *et al.* 2014), as expected, given that *F. albicollis* was used as reference for mapping. The number of Ns in the assembly was 19%, and the total GC content was 31% (AT = 50%). All statistics of the chiffchaff assembly are presented in Table S2 in File S1. The assessment of the chiffchaff assembly completeness based on conserved gene sets showed that 80.9% of vertebrate and 50.3% of eukaryotic genes were completely covered. The fractions of fragmented genes in the vertebrate and eukaryotic gene sets were 6.5 and 4.9%, respectively, and missing genes constituted 12.6 and 44.8% of the gene sets (Table S3 in File S1). For comparison, the high-quality *F. albicollis* and *C. corone* assemblies cover only slightly higher fractions of complete vertebrate (92.8 and 84.4%, respectively) and eukaryotic (50.3% in both species) genes, and have a slightly lower proportion of completely missing genes; 2.5 and 10.1% in the vertebrate gene set (Table S3 in File S1).

### Global estimates of genetic diversity and differentiation

**Figure 1:**
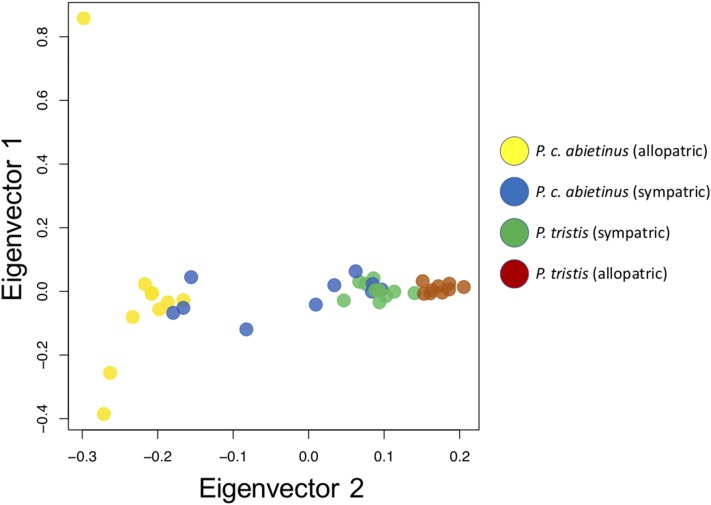
A visual illustration of genetic similarities across all chiffchaff individuals using a PCA as implemented in the R Bioconductor package SNPRelate (Zheng 2012) of 220,097 unlinked (pairwise LD as estimated by *r^2^* < 0.2) high quality SNPs. Samples are color-coded according to origin of individuals (yellow, allopatric *abietinus*; blue, sympatric *abietinus*; green, sympatric *tristis*; brown, allopatric *tristis*). Axes show the eigenvalues from the PCA for the two components explaining most of the variation (PC1 = 4.6% and PC2 = 3.2%).

**Figure 2:**
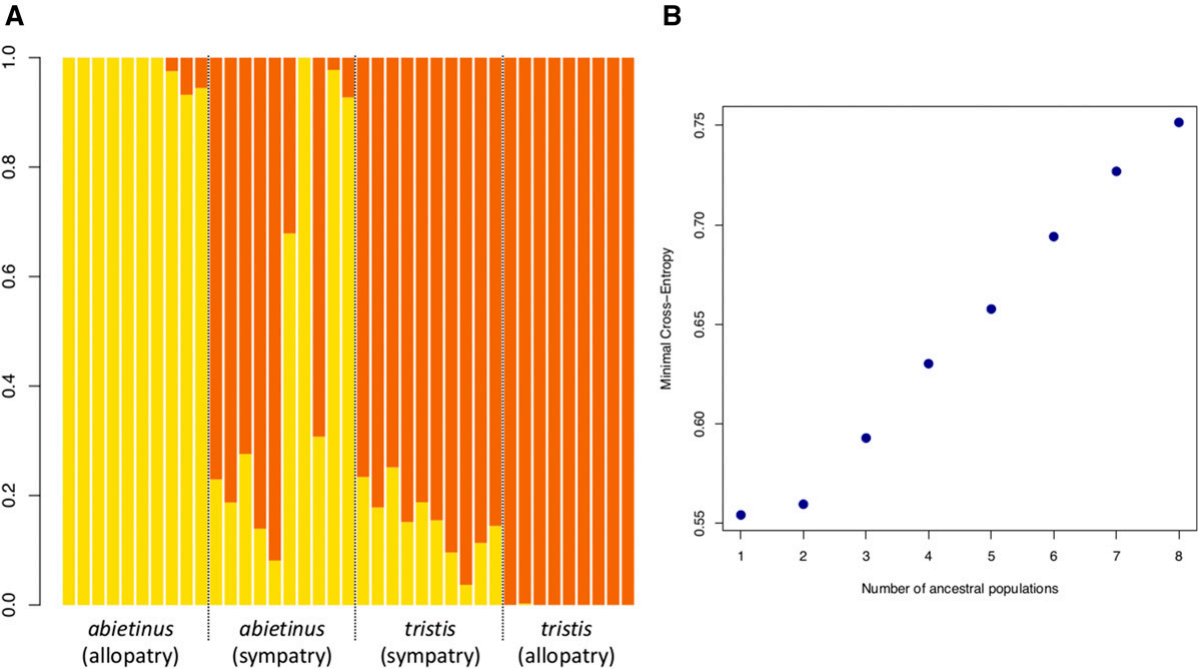
Summary of the analysis of sample clustering using the R Bioconductor package LEA with 220,097 high quality SNPs (Frichot 2014, 2015). (A) Yellow, *abietinus*-derived alleles; brown, *tristis* derived alleles. Each vertical bar represents one individual, and samples are sorted according to population origin with allopatric *abietinus* to the far left, sympatric populations in the middle, and allopatric *tristis* to the far right. (B) Summary of error (entropy) rates for *K* = 1–8 predefined populations. The largest rate change in entropy occurs from two to three populations indicating that *K* = 2 is the optimal number of clusters (Frichot 2014, 2015).

In total, we detected ∼7 million SNPs across all individuals from both species, of which 6,912,505 were scored in the allopatric population pairs and 6,843,490 in the sympatric population pairs (Table 1). We observed a significantly higher proportion of shared SNPs (sympatry = 48.0%, allopatry = 35.5%), and a lower proportion of fixed differences (sympatry = 0.0%, allopatry = 0.3%) between *abietinus* and *tristis* in sympatry than in allopatry (Chi-Square test: χ^2^ = 283,510, *df* = 3, p-value < 2.2 × 10^−16^; Table 1). The proportion of fixed differences on the Z-chromosome was significantly higher in the comparison of allopatric *abietinus* and *tristis* (5.4%), but there were no fixed differences on the Z-chromosome in the sympatric comparison (Chi-Square test: χ^2^ = 198,380, *df* = 3, p-value < 2.2 × 10^−16^; Table 1). Both the PCA- and the structure (sNMF) analysis clearly separated allopatric *abietinus* and *tristis* (Figure 1 and Figure 2) with individuals from the sympatric regions clustering at the intermediate space in the PCA analysis (Figure 1), and showing considerable admixture proportions in the sNMF analysis (Figure 2). In the PCA, allopatric *abietinus* showed more interindividual variation than allopatric *tristis*, and sympatric *tristis* tended to cluster closer with allopatric *tristis*, while sympatric *abietinus* were more scattered across the range between the allopatric groups (Figure 1). This pattern was consistent with the clustering analysis where allopatric *abietinus* and *tristis* form clearly separated groups (optimal *K* = 2), and all sympatric *tristis* had >75% of the assigned proportions shared with allopatric *tristis* while sympatric *abietinus* varied from being assigned with 100% to *abietinus* to >90% with *tristis* (Figure 2).

**Table 1:** Summary of numbers (#) and proportions (in %) of shared, private, and fixed polymorphisms across allopatric and sympatric *abietinus* (*ab*.) and *tristis* (*tr*.) for the autosomes (A), the Z-chromosome (Z), and all chromosomes jointly (All)

Region (Chrom)	# Shared	# Private *ab*.	# Private *tr*.	Fixed	# Total
Allopatry (A)	2,426,608 (36.0%)	2,687,834 (39.9%)	1,614,661 (24.0%)	l9,505 (0.1%)	6,738,608
Sympatry (A)	3,188,645 (48.0%)	1,884,193 (28.3%)	1,574,541 (23.7%)	0 (0.0%)	6,647,379
Allopatry (Z)	27,559 (15.8%)	83,921 (48.3%)	52,973 (30.5%)	9,444 (5.4%)	173,897
Sympatry (Z)	95,951 (48.9%)	57,463 (29.3%)	42,697 (21.8%)	0 (0.0%)	196,111
Allopatry (All)	2,454,167 (35.5%)	2,771,755 (40.1%)	1,667,634 (24.1%)	18,949 (0.3%)	6,912,505
Sympatry (All)	3,284,596 (48.0%)	1,947,656 (28.4%)	1,617,238 (23.6%)	0 (0.0%)	6,843,490

**Figure 3:**
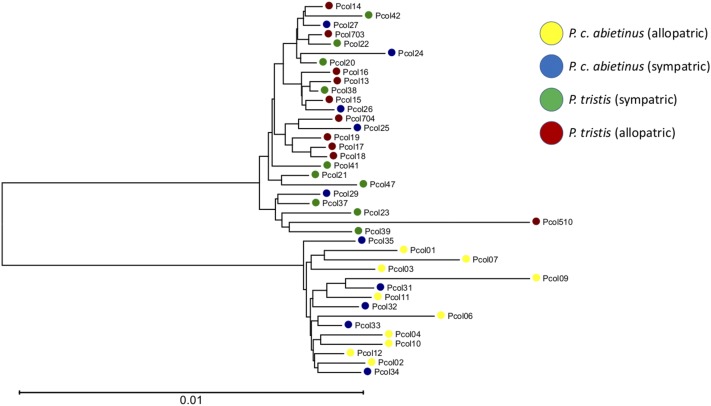
An unrooted phylogeny representing the entire mitochondrial genome for all 40 samples included in the study. The length of the scale bar represents 0.01 substitutions per site, and nodes are color-coded according to origin of populations (yellow, allopatric *abietinus*; blue, sympatric *abietinus*; green, sympatric *tristis*; brown, allopatric *tristis*).

The mtDNA genome analyses revealed two distinct mtDNA clades predominantly reflecting differentiation between allopatric *abietinus* and *tristis*, but with considerable nesting of sympatric *abietinus* individuals within the *tristis* clade (Figure 3 and Figure S2 in File S1). Allopatric *tristis* clustered in two separate groups, birds from Southern Siberia being in a derived clade while birds from Central and Eastern Siberia and the Ural Mountains were scattered in basal lineages in the “*tristis*”-type mtDNA group, and birds from the Southern sympatric zone showed higher diversity in mtDNA haplotypes than birds from the Northern sympatric region (Figure S2 in File S1). The analyses of mtDNA genetic variation within and between populations showed that diversity was higher in *abietinus* than in *tristis* in allopatry (*Ɵ_π_* = 5.4 ± 0.3 × 10^−3^ *vs.* 4.0 ± 0.3 × 10^−3^ for *abietinus* and *tristis*, respectively), and the pattern was even more pronounced in sympatry (*Ɵ_π_* = 12.6 ± 0.5 × 10^−3^ *vs.* 3.7 ± 0.3 × 10^−3^ for *abietinus* and *tristis*, respectively). MtDNA genetic divergence was also higher between allopatric than between sympatric *abietinus* and *tristis* (Maximum Likelihood corrected numbers of substitutions per site = 0.0213 ± 0.0008 and 0.0117 ± 0.0004 in allopatry and sympatry, respectively, Table S4 in File S1). Overall, this picture a scenario where *abietinus* and *tristis* diverged in allopatry roughly 1–2 MYA (following the rationale behind a body-size corrected mtDNA genome divergence rate as proposed by Nabholz *et al.* 2016), and subsequently came into secondary contact in the current sympatric region where hybridization and backcrossing resulted in higher levels of allele sharing between species, with higher rates of introgression from *tristis* into *abietinus*.

There was no striking difference in nuclear genetic diversity across populations although *abietinus* showed slightly higher global genetic diversity than *tristis* both in allopatry and sympatry (average *Ɵ_π_* in allopatric and sympatric *abietinus* = 0.0053 ± 0.0031 and 0.0051 ± 0.0029, respectively and 0.0047 ± 0.0031 and 0.0049 ± 0.0030 in allopatric and sympatric *tristis*, Table 2). As expected given a lower *N_e_*of sex-chromosomes than of autosomes, both species had lower genetic diversity on the Z-chromosome (average *Ɵ_π_* = 0.0031 ± 0.0035 and 0.0025 ± 0.0030, for allopatric *abietinus* and *tristis*, respectively) as compared to the autosomes combined (*Ɵ_π_* = 0.0054 ± 0.0030 and 0.0048 ± 0.0030) – the same pattern was observed in sympatry (Table 2). Since demographic history may affect allele frequency distributions and patterns of LD, we assessed if further understanding of the underlying processes behind allele sharing in sympatry could be gained by using standard population genetic summary statistics (Tajima’s *D*) and linkage information. In summary, the global estimates of Tajima’s *D* were generally negative, in line with a bottleneck + expansion model for all populations, but less pronounced in allopatric *tristis* (Table 2). There were only minor differences in global decay patterns of LD across populations (Figure S3 in File S1 and Table 2). The history of the two species provides a starting point for exploring patterns of genetic differentiation across the genome as a consequence of secondary contact and recurrent hybridization, and to investigate how gene-flow affects global and regional genomic differentiation and characterize regions that may be shielded from introgression.

**Table 2:** Summary of intrapopulation nuclear genomic pair-wise nucleotide diversity (*Ɵ_π_*), Watterson’s theta (*Ɵ_W_*), Tajima’s *D* (*T_D_*), and LD (*r^2^*, per base pair) as estimated in nonoverlapping 10 kb windows across the chiffchaff genome

Population	*Ɵ_π_*	*Ɵ_W_*	*T_D_*	*r^2^*
Autosomes				
Allopatric *abietinus*	0.0054 ± 0.0030	0.0064 ± 0.0025	−0.68 ± 0.37	0.029 ± 0.049
Allopatric *tristis*	0.0048 ± 0.0030	0.0051 ± 0.0025	−0.28 ± 0.39	0.030 ± 0.053
Sympatric *abietinus*	0.0052 ± 0.0029	0.0060 ± 0.0024	−0.58 ± 0.36	0.028 ± 0.052
Sympatric *tristis*	0.0050 ± 0.0029	0.0056 ± 0.0024	−0.50 ± 0.39	0.029 ± 0.055
Z-chromosome				
Allopatric *abietinus*	0.0031 ± 0.0035	0.0038 ± 0.0027	−0.85 ± 0.45	0.022 ± 0.062
Allopatric *tristis*	0.0025 ± 0.0030	0.0028 ± 0.0024	−0.59 ± 0.49	0.025 ± 0.080
Sympatric *abietinus*	0.0034 ± 0.0027	0.0039 ± 0.0022	−0.50 ± 0.41	0.023 ± 0.057
Sympatric *tristis*	0.0030 ± 0.0029	0.0035 ± 0.0023	−0.66 ± 0.37	0.022 ± 0.061

All summary statistics are global averages for autosomes and the Z-chromsome, respectively, with SD estimated from variance across windows.

**Figure 4:**
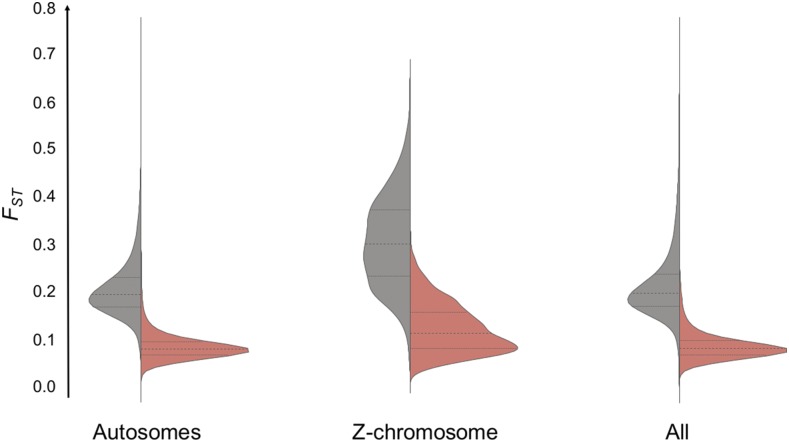
Violin plots showing the distributions of genetic differentiation (*F_ST_*) on the Z-chromosome, autosomes and all chromosomes combined in comparisons of allopatric (gray) and sympatric (red) *abietinus* and *tristis* pairs. Horizontal solid lines within distribution curves indicate means, and broken lines the SD across all 10-kb windows in the genome.

**Figure 5:**
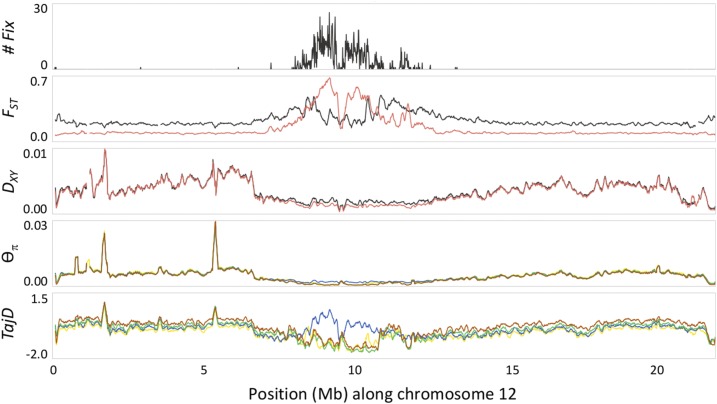
Illustration of regional variation in fixed differences (allopatric comparison only, top panel, *# Fix*), genetic differentiation (second panel, *F_ST_*), absolute divergence (middle panel, *D_XY_*), nucleotide diversity (fourth panel, *Ɵ_π_*) and Tajima’s *D* (bottom panel, *TajD*) represented by chromosome 12. Values on *y*-axes represent ranges of each respective parameter and colors indicate species comparison (gray, allopatry; red, sympatry for *# Fix*, *F_ST_* and *D_XY_*) or population (yellow, allopatric *abietinus*; brown, allopatric *tristis*; blue, sympatric *abietinus*; green, sympatric *tristis* for Ɵ_π_ and *TajD*).

**Figure 6:**
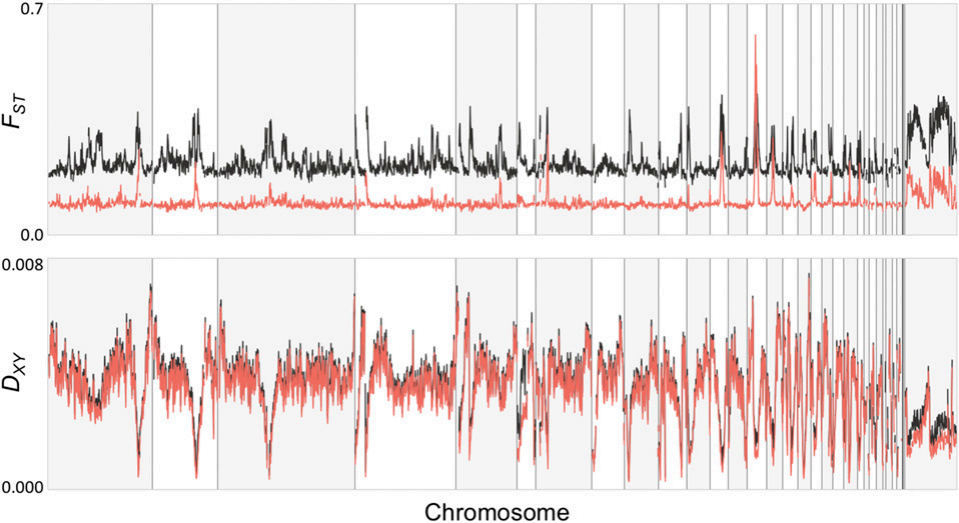
Illustration of the variation in genetic differentiation (*F_ST_*, top panel) and absolute genetic divergence (*D_XY_*, bottom panel) across the genome between allopatric (gray line) and sympatric (red line) *abietinus* and *tristis*. Chromosomes are ordered from chromosome 1 to chromosome Z, and every other chromosome is shaded in light gray.

The global level of genetic differentiation between species was significantly lower in the sympatric region (mean ± SD of *F_ST_* across windows = 0.099 ± 0.038) than in the allopatric regions (0.220 ± 0.064) (Wilcoxon’s test: *W* = 1.04 × 10^10^, p-value <2.2 × 10^−16^), and the level of genetic differentiation was significantly higher on the Z-chromosome than on the autosomes (only allopatric comparison shown: Wilcoxon’s test: *W* = 1.05 × 10^8^, p-value < 2.2 × 10^−16^; Figure 4 and Table 3). An opposite pattern was observed for absolute divergence (*D_XY_*), with reduced divergence on the Z-chromosome as compared to autosomes [Wilcoxon’s test: *W* = 1.17 × 10^8^, p-value < 2.2 × 10^−16^ (allopatry); *W* = 1.0 × 10^8^, p-value < 2.2 × 10^−16^ (sympatry)], although the quantitative difference between allopatry and sympatry was less pronounced [global *D_XY_* = 0.0085 ± 0.0058 (allopatry) and 0.0081 ± 0.0056 (sympatry); Wilcoxon’s test: *W* = 5.76 × 10^9^, p-value < 2.2 × 10^−16^], except for the Z-chromosome where *D_XY_* was considerably reduced in sympatry as compared to allopatry [Z-chromosome *D_XY_* = 0.0061 ± 0.0039 (allopatry) and 0.0055 ± 0.0034 (sympatry); Wilcoxon’s test: *W* = 5.1 × 10^9^, p-value < 2.2 × 10^−16^; Table 3). The overall level of genetic differentiation was hence higher on the Z-chromosome than on the autosomes, both in allopatry and sympatry, but the opposite was observed for absolute divergence (Figure 4, Figure 5, and Figure 6).

**Table 3:** Summary of the levels of genetic differentiation (*F_ST_*) and absolute divergence (*D_XY_*) across allopatric and sympatric *abietinus* and *tristis* for the autosomes (A), the Z-chromosome (Z), and all chromosomes jointly (All)

Region (chrom)	*F_ST_*	*D_XY_*
Allopatry (A)	0.214 ± 0.057	0.0086 ± 0.0059
Sympatry (A)	0.097 ± 0.036	0.0083 ± 0.0056
Allopatry (Z)	0.313 ± 0.093	0.0061 ± 0.0039
Sympatry (Z)	0.133 ± 0.051	0.0055 ± 0.0034
Allopatry (All)	0.220 ± 0.064	0.0085 ± 0.0058
Sympatry (All)	0.099 ± 0.038	0.0081 ± 0.0056

All summary statistics are averages from windows within each respective chromosome class with SD estimated from variance across windows.

### Regional variation in genetic diversity and differentiation

**Figure 7:**
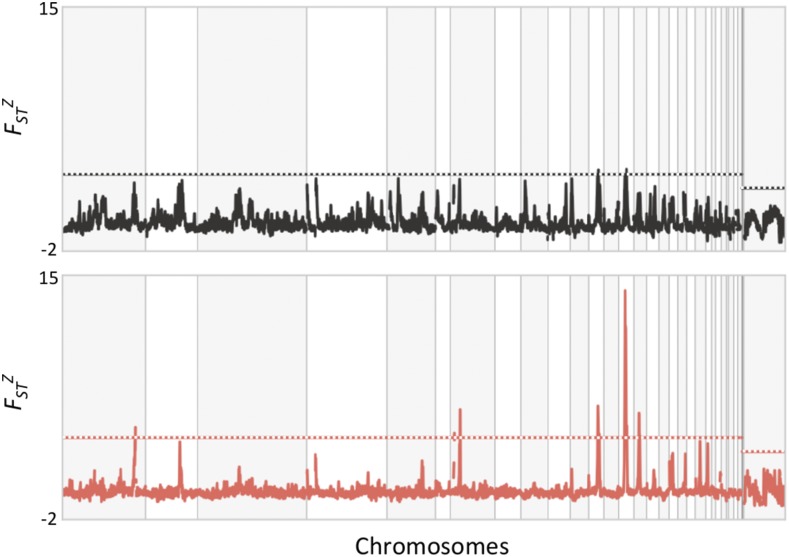
Estimates of relative differentiation (*F_ST_^Z^*) between *abietinus* and *tristis* for 10 kb windows across the genome. The upper 99th percentiles are represented by horizontal barred lines for the allopatric (top panel, gray) and sympatric (bottom panel, red) comparisons, respectively. Regions with significantly higher density of outlier windows are indicated with gray (allopatry) and red (sympatry) solid blocks.

Global estimates of nuclear genetic diversity showed highly heterogeneous patterns across the genome of both species, both in allopatric and sympatric populations (Figure 5, Figure 6, and Figure S4 in File S1). Similar to many previous studies of avian (and other) species pairs, the level of genetic differentiation varied extensively across the genome, with presence of distinct *F_ST_* “peaks” in both the allopatric and sympatric *abietinus* and *tristis* species comparisons (Figure 6 and Figure S4 in File S1). A striking difference between allopatry and sympatry was that the overall level of differentiation (but not absolute divergence) was lower, and the differentiation peaks were narrower and more pronounced in the sympatric comparison (Figure 6, Figure 7, and Figure S4 in File S1), resulting in significantly different *F_ST_*-distributions (Kolmogorov-Smirnov test: *D* = 0.88, p-value < 2.2 × 10^−16^). There was a significant negative correlation between the level of genetic differentiation and genetic diversity in both populations [Pearson’s *r* = −0.29 and −0.32 for *abietinus* and *tristis*; p-value < 2.2 × 10^−16^ in both tests (allopatry); *r* = −0.17 and −0.21; p-value < 2.2 × 10^−16^ in both tests (sympatry)], and also a negative correlation between differentiation and absolute divergence, both in allopatry (Pearson’s *r* = −0.225, *t* = −74.421, *df* = 103,770, p-value < 2.2 × 10^−16^) and in sympatry (Pearson’s *r* = −0.130, *t* = −30.823, *df* = 103,630, p-value < 2.2 × 10^−16^ ; Figure S5 in File S1). Tajima’s *D* was, in general, more negative in regions of high differentiation in comparison to the remaining parts of the genome (Pearson’s *r* = −0.070 to −0.166, p-values < 2.2 × 10^−16^), with the exception of sympatric *abietinus* that showed a positive correlation between Tajima’s *D* and the level of genetic differentiation (Pearson’s *r* = 0.162, p-value < 2.2 × 10^−16^; Figure 5, Figure S4, and Figure S6 in File S1). The analysis of the levels of genetic diversity and absolute divergence shows that the correlation is very strong, both in allopatry and in sympatry, with only little deviation from the linear relationship between *Ɵ_π_* and *D_XY_* (Figure S7 in File S1). We observed a higher variance in absolute divergence in allopatry than in sympatry, and a more pronounced clustering of high differentiation windows at the lower left end in the graph (low genetic diversity and low absolute divergence) in autosomes than for the Z-chromosome (Figure S7 in File S1).

Windows with *F_ST_^Z^* values higher than the 99th percentile in the distributions were highly clustered in specific regions for both the allopatric and the sympatric comparison (Figure 7). We tested for significant clustering by scanning the genome for regions where blocks of 50 windows (each window was 10 kb long) contained 10 or more *F_ST_^Z^* outlier windows (the probability of 10 or more outliers appearing in a 50-window block was estimated to 7.13 × 10^−11^ if outliers are assumed to be randomly scattered across the genome). In the allopatric comparison, 19 blocks representing 13 chromosomal regions (chromosomes 1, 1A, 2, 3, 4, 5, 7, 8, 9, 10, 12, and 28), were identified as significantly enriched for *F_ST_^Z^* outliers, and the analogous analysis in sympatry revealed 13 windows, representing 10 different regions located on chromosomes 1, 1A, 5, 10, 12, 13, 17, 20, and 21 (Figure 7 and Table S5 in File S1). The identified regions were highly overlapping between the two different comparisons, but, again, regions were narrower and more pronounced in sympatry (Figure 7). The chromosome regions with high density of *F_ST_^Z^* outliers contained 238 and 274 protein coding genes in the allopatric and the sympatric comparisons, respectively (Table S6 in File S1). The GO enrichment analysis revealed no over-representation of specific terms for genes in differentiation outliers in allopatric comparison (p-value threshold 0.01 after correcting for multiple testing), and only one term associated with regulation of cellular component organization in the sympatric comparison for two partly overlapping gene sets (Table S6 in File S1)

### Protein coding genes in differentiation peaks

Global genomic differentiation was significantly lower in sympatry than in allopatry with more pronounced and narrower differentiation peaks, indicating recurrent gene-flow in large portions of the genome of the two species. The highly differentiated regions in sympatry may indicate that these regions have experienced restricted gene-flow, and therefore we wanted to identify potential targets of diversifying selection and/or incompatibility within these regions. Omitting the Z-chromosome, which, in general, showed elevated differentiation across the entire chromosome (Figure 6), and no relative differentiation outliers (Figure 7), we found 26 protein coding genes in the 10 kb windows with the highest level of genetic differentiation nested within blocks of high density of relative differentiation outliers (see above) in the comparison of sympatric *abietinus* and *tristis* (Table 4). Among the 26 genes, three were uncharacterized, and the remaining 23 had diverse functions, mainly related to ion transport, gene regulation (transcription factors, histone binding, and phosphorylation), muscle development, learning (*ARF4*), sensory perception of sound (*OTOG*), and pigmentation (*HPS5*) (Table 4).

**Table 4:** Protein coding genes identified in the 10-kb windows with the highest genetic differentiation (*F_ST_*) nested within the chromosome regions enriched for windows with high relative differentiation (*F_ST_^Z^*) between sympatric *abietinus* and *tristis*

Gene Name/Ensemble ID	Chromosome	Annotation/Function
*EMSY (C11orf30)*	1	Transcription factor
*ATF7IP*	1A	Transcription factor
*PLBD1*	1A	Phospholipase B domain
*GUCY2C*	1A	GMP biosynthetic process, phosphorylation
*SLC9A3*	1A	Sodium ion transport
*TPPP*	2	Microtubule bundle formation (brain)
*BRD9*	2	Nucleic acid/histone binding; gene regulation
*COL15A1*	2	Cell adhesion
*ENSFALG00000013290*	2	Novel transcript
*ENSFALG00000006425*	3	Signal transduction/response to stimulus
*DZANK1*	3	Mitophagy
*ANO5*	5	Chloride transport
*SLC17A6*	5	Transmembrane transport
*LUZP2*	5	Leucine zipper 2 protein
*PLEKHA7*	5	Epithelial cell-cell adhesion
*ABCC8*	5	Transcription factor
*OTOG*	5	Sensory perception of sound
*HPS5*	5	Pigmentation/organelle organization
*SERGEF*	5	Negative regulation of protein secretion
*KCNC1*	5	Potassium ion transport
*MYOD1*	5	Muscle tissue development
*ENSFALT00000008878*	12	Microtubule activity
*PDE12*	12	Phosphodiesterase 12
*ARF4*	12	Cell cilia function/signaling/rhodopsin
*DENND6A*	12	Regulation of cell-cell adhesion
*SLMAP*	12	Muscle contraction

Genes are ordered according to position in the genome. The function is inferred from GO terms (biological process and/or molecular function) as indicated in Ensembl (http://www.ensembl.org/Ficedula_albicollis/Gene/Ontologies).

### Functional relevance of fixed, shared, and private polymorphisms

**Figure 8:**
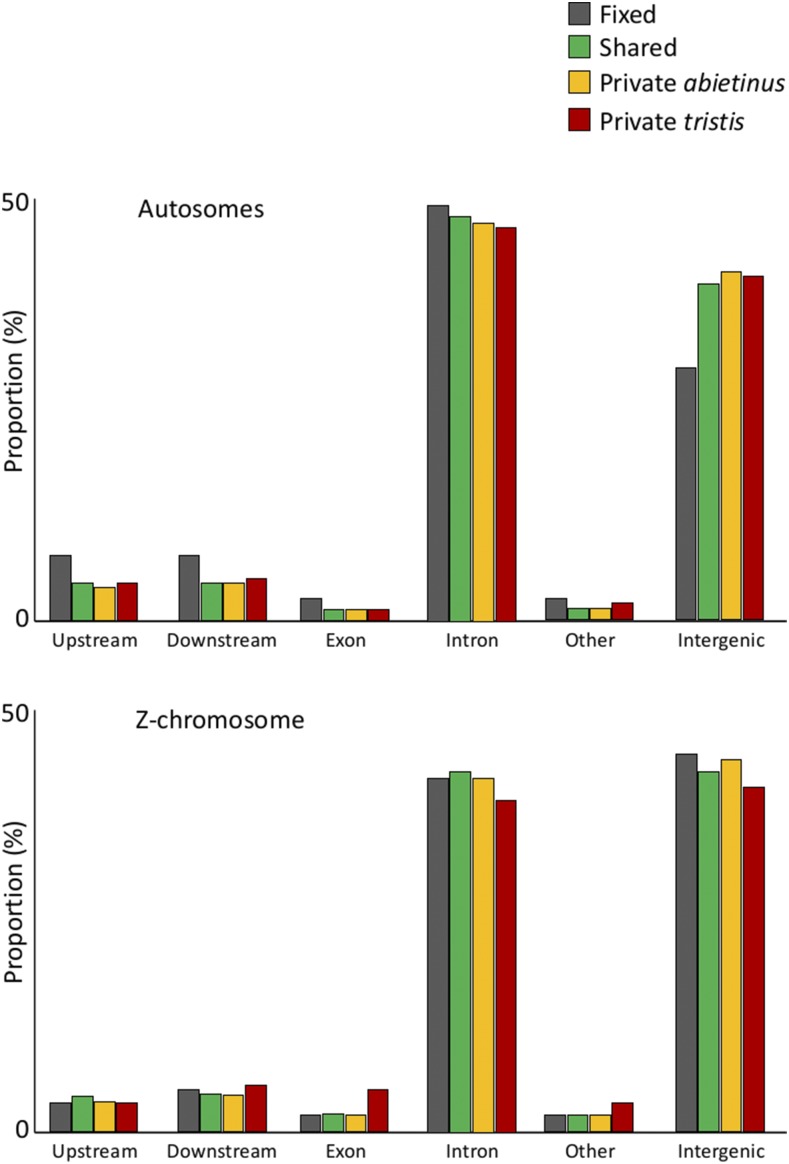
Bar plots showing the relative frequencies of shared (*n* = 1,224,635; green), private [yellow (*n* = 734,710) and brown (*n* = 843,724) for *abietinus* and *tristis*, respectively) and fixed (*n* = 7609; gray) polymorphisms between allopatric *abietinus* and *tristis*. SNPs on the autosomes are presented in the top panel, and Z-chromosome linked SNPs are presented in the bottom panel.

To obtain further information about the underlying forces generating a heterogeneous distribution of genetic diversity and differentiation, we assessed the functional relevance of different categories of SNPs. This analysis was focused on the allopatric comparison, only since we did not identify any fixed differences between sympatric *abietinus* and *tristis*, and we applied more stringent filtering (sequence coverage of at least 5× per individual for at least nine individuals in both allopatric *abietinus* and *tristis*) to minimize the risk of including erroneously called fixed differences in this analysis. Among all high-quality SNPs selected for this analysis (2,810,678 SNPs), we found that almost half of them (43.6%) were shared polymorphisms, while only 7609 SNPs (0.27%) represented fixed differences. We generated annotation information for all SNPs from all of the eight SNP sets (shared, private *abietinus*, private *tristis*, and fixed differences for the autosomes and the Z-chromosome, respectively) using the annotation available for the *F. albicollis* genome assembly (http://www.ensembl.org/Ficedula_albicollis/Info/Index). For autosomes, there was an excess in the proportion of fixed differences in genes, or chromosome regions in the vicinity of genes, while there was an underrepresentation of fixed differences in intergenic sequences, but this pattern was not present on the Z-chromosome (Figure 8). Since fixed differences are overrepresented in high differentiation regions (Figure 5 and Figure S6 in File S1), this indicates that patterns of elevated differentiation cannot entirely be explained by restricted recombination and less efficient selection, but also that diversifying selection has driven certain alleles to fixation between *abietinus* and *tristis*. We therefore took the analysis one step further by classifying all the fixed differences between the species in allopatry. The results showed that of the 7609 fixed differences identified, the majority were as expected located in introns, intergenic regions or noncoding regions adjacent to genes (*n* = 7458), but we also identified 134 exonic fixed differences corresponding to 116 synonymous and 18 nonsynonymous positions, and 17 fixed differences potentially affecting splice signals. The genes associated with the fixed differences at nonsynonymous positions or splice sites are all potential candidates for underlying species-specific characteristics of *abietinus* and *tristis*, respectively, and are presented in Table S7 in File S1. Two of the genes had more than one nonsynonymous substitution, and, based on functional studies in other organisms, these may be involved in the innate immune response (*FYB*) and lipid metabolism (*PRUNE2*). Another two genes had both nonsynonymous and splice altering differences, and these genes have functions related to gene expression regulation via histone methylation (*KDM4C*), microtubule activity, and intracellular transport (*KIF24*).

## Discussion

Shipilina *et al.* (2017) used nuclear SNP information from one of the sequencing batches and restriction data from a single mtDNA gene sequence (*CYTB*) in the same set of chiffchaff samples. They investigated population structure in *abietinus* and *tristis* in relation to phenotypic variation in allopatry and sympatry, and verified that the intermediate phenotypes and mixed singers observed in the area around the Ural mountains represented admixed individuals rather than a third taxon previously suggested to be present in the area (Shipilina *et al.* 2017). Here, we went one step further by developing a reference-assisted genome assembly, and using the complete mtDNA genome and higher coverage nuclear genomic resequencing data to generate quantitative information about genome-wide patterns of regional variation in genetic polymorphism, divergence, and differentiation. The polymorphism data were used to investigate how hybridization in secondary contact affects patterns of genome differentiation and to identify regions under diversifying selection, potentially involved in reproductive barriers between the species. The chiffchaff assembly covered ∼81% of the genome (as estimated from comparison to the collared flycatcher, *F. albicollis*), and had sufficient quality to use as a backbone for mapping reads from resequencing efforts involving allopatric and sympatric populations of *abietinus* and *tristis*. The level of complete and partial coverage of conserved gene sets was, for example, similar to high-quality avian genome assemblies (*F. albicollis* and *C. corone*).

Initially, we used the new polymorphism data to assess population structure and genomic variation within and between *abietinus* and *tristis* to have a thorough background for more detailed analyses of genomic regions that showed patterns of differentiation consistent with diversifying selection and/or genetic incompatibilities. Our estimates of genetic diversity, genetic differentiation, allele frequency distributions, LD, and population structure for both mtDNA and nuclear DNA supported the scenario suggested by Shipilina *et al.* (2017). The two species were most likely isolated in allopatry and accumulated novel variants and allele frequency changes, which was followed by secondary contact and interspecific gene-flow when distribution ranges expanded, resulting in reduced global differentiation (Shipilina *et al.* 2017; Marova *et al.* 2013). We observed only minor differences in the levels of genetic diversity across populations, with allopatric *abietinus* showing slightly higher proportion of private polymorphisms and a higher genetic diversity, but a higher proportion of low frequency variants (more negative Tajima’s *D*) than allopatric *tristis*, despite the smaller sampling range of *abietinus* than *tristis* (Shipilina *et al.* 2017). This may reflect a more severe or longer bottleneck, and a less dramatic expansion in *tristis* during and after glacial cycles. In comparison to a well-studied avian study system with potentially similar demographic histories—the pied (*Ficedula hypoleuca*) and the collared flycatcher (*F. albicollis*), where average nucleotide diversity ranges from 3.2 × 10^−3^ to 4.0 × 10^−3^ (Burri *et al.* 2015)—the diversity levels were 10–20% higher in chiffchaff, suggesting higher long-term effective population sizes, potentially as a result of less dramatic contractions during glacial maxima. In sympatry, there was a reduction in the fraction of private polymorphisms, which was more pronounced in *abietinus* than in *tristis*, and the mtDNA divergence in intraspecific comparisons of allopatric and sympatric populations was higher in *abietinus* than in *tristis*. Furthermore, sympatric *abietinus* had a higher Tajima’s *D*, indicating a lower proportion of low frequency alleles. This suggests that sympatric *abietinus*-like birds also harbor *tristis* nuclear and mtDNA alleles, which is in line with previous analyses (Shipilina *et al.* 2017). Taken together, this portrays a scenario where secondary contact and recurrent hybridization and back-crossing has generated a reduction in genome-wide differentiation in sympatry, and that the external appearance of an individual does not necessarily reflect genomic composition. Interestingly, this indicates that individual phenotype is not dependent on the effect of many genes scattered throughout the genome, but rather that relatively few genes may underlie the observed differences between the species. Having this background knowledge provides a good position from which to investigate regional variation in diversity, differentiation, and divergence to draw conclusions on specific processes underlying the observed patterns in allopatry and sympatry.

There was a considerable reduction in genetic diversity (*Ɵ_π_*) on the Z-chromosome as compared to the autosomes in both *abietinus* and *tristis* and the level of genetic differentiation (*F_ST_*) was on average higher on the Z-chromosome in both allopatry and sympatry. Interestingly, the level of absolute divergence (as estimated by *D_XY_*) was considerably reduced on the Z-chromosome as compared to the autosomes, similar to previous observations in the greenish warbler species complex (Irwin *et al.* 2016). This indicates that lower postdivergence gene-flow has not been the main force affecting the increase in differentiation on the Z-chromosome, but rather that selection in the ancestral lineage has resulted in reduced diversity prior to the split between *abietinus* and *tristis* (Cruickshank and Hahn 2014; Nachman and Payseur 2012; Irwin *et al.* 2016). The overall elevated level of differentiation on the Z-chromosome is a frequently observed pattern (Burri *et al.* 2015; Ellegren *et al.* 2012; Irwin *et al.* 2016; Wolf and Ellegren 2017; Oyler-McCance *et al.* 2015); however, several characteristics of the sex-chromosomes (Vicoso and Charlesworth 2006) contribute to difficulties in the interpretation of the exact mechanisms underlying the observed patterns on the Z-chromosome. First, the Z-chromosome does not recombine in females (except for the pseudoautosomal region, PAR), and, therefore, it is expected that the overall recombination rate will be reduced on the Z-chromosome as compared to the autosomes of similar size, which do recombine in both males and females (Irwin *et al.* 2016; Qvarnström and Bailey 2009). This results in lower efficiency of selection in general (reducing *N_e_* and increasing the effect of drift), and should also increase the effect of linked selection on the Z-chromosome. Second, it is plausible that the Z-chromosome harbors a gene set that is nonrepresentative for the genome as a whole, *e.g.*, enriched for genes involved in local adaptation or reproductive isolation, as has been suggested for the X-chromosome in *Drosophila* (Presgraves 2008). We know, for example, from previous studies in *Ficedula* flycatchers, that the Z-chromosome plays a particularly important role for traits under sexual selection and species recognition (Ellegren *et al.* 2012; Qvarnström and Bailey 2009; Qvarnström *et al.* 2010; Sætre and Sæther 2010), and this could potentially be translated also to the situation in chiffchaff, since both song and plumage are important signals for mate choice and species recognition in *Phylloscopus* warblers (Lyu *et al.* 2016; Arvidsson and Neergaard 1991), including *abietinus* and *tristis* (Marova *et al.* 2017). Third, the effective population size (*N_e_*) of the Z-chromosome is reduced as compared to autosomes. With an equal sex-ratio, *N_e_* for the Z-chromosome is 3/4 (75%) of the *N_e_* for autosomes, since females only have a single Z-chromosome (in birds, male is the homogametic sex and harbors two Z chromosomes, and female is the heterogametic sex with one Z and one W chromosome). In birds, males also generally have higher variance in reproductive output than females, further reducing the effective population size of the Z-chromosome and increasing the effect of genetic drift. Fourth, since the Z-chromosome is in hemizygous state in female birds, recessive mutations are expressed in females, and this can contribute to both faster evolution (Vicoso and Charlesworth 2009; Sackton *et al.* 2014; Mank *et al.* 2007, 2010) and more pronounced antagonistic effects across sexes, leading to sex-biased expression patterns (Mank 2009; Ellegren and Parsch 2007). Finally, since the Z-chromosome spends 2/3 of the time (in an evolutionary timescale perspective) in the male germline, it is possible that mutation rates are higher on the Z-chromosome than on the autosomes due to male-biased mutation (Ellegren and Fridolfsson 1997; Hurst and Ellegren 1998; Wilson Sayres and Makova 2011). The observed patterns in chiffchaff suggest that the most plausible explanation is that genetic drift is a major force affecting allele frequency changes on the Z-chromosome, and that directional selection affects larger regions due to linkage, and these forces have increased differentiation across the entire chromosome. Mutation bias, however, probably has had only a minor effect (if any), since absolute divergence is considerably reduced as compared to the autosomes. In addition, selection has likely been a key force reducing overall diversity on the Z-chromosome prior to the split between *abietinus* and *tristis*, and followed by accumulation of allele frequency changes due to strong effects of drift as a consequence of combined effects of the specific features of the Z-chromosome listed above. Hence, there is no evidence in our allele frequency data for more restricted gene-flow on the Z-chromosome, which is also supported by the fact that no fixed differences were observed on the Z-chromosome when comparing *abietinus* and *tristis* in the sympatric zone.

Similar to the observations when comparing the Z-chromosome to the autosomes, the highly differentiated regions across chiffchaff species in general showed reduced diversity and absolute divergence. There was also a strong correlation between absolute divergence and genetic diversity. Again, similar to the situation for the Z-chromosome, this may be translated to a scenario where linked selection and regional lowered effective population size has resulted in loss of genetic variation prior to the divergence of the two lineages (Cruickshank and Hahn 2014; Nachman and Payseur 2012). The levels of differentiation are expected to vary regionally as a consequence of stochastic allele frequency changes, also when the level of selection is equal across regions in the genome, especially if populations are structured (Baird 2017; Lohse 2017). However, we observed very distinct and narrow differentiation peaks, especially in sympatry, which indicates that additional factors affect the differentiation landscape between chiffchaff species. One plausible explanation is that the recombination rate varies considerably across regions in the chiffchaff genome, similar to what has been observed in other bird species (Kawakami *et al.* 2014; Singhal *et al.* 2015; Backström *et al.* 2010). This leads to reduced effective population size in regions with low recombination rate, allowing for more rapid allele frequency shifts across species due to genetic drift in those regions (Burri *et al.* 2015; Burri 2017a,b; Ellegren and Wolf 2017; Ravinet *et al.* 2017; Cruickshank and Hahn 2014; Nachman and Payseur 2012; Noor and Bennett 2009). We do not have access to recombination data for the chiffchaff, but several recent studies indicate that recombination landscapes may be conserved across divergent avian lineages (Singhal *et al.* 2015), leading to similar patterns of genetic diversity and differentiation across independent species pair comparisons (Burri *et al.* 2015; Irwin *et al.* 2016; Dutoit *et al.* 2017; Van Doren *et al.* 2017; Vijay *et al.* 2017). In support of that, when visually comparing the location of differentiation islands identified in the chiffchaff, we see a considerable overlap with for example differentiation islands in *Ficedula* flycatchers (Burri *et al.* 2015; Ellegren *et al.* 2012). Hence, although we acknowledge that demographic history definitely affects differentiation patterns, our data suggest that the highly heterogenic landscape of genome differentiation between *abietinus* and *tristis* can be traced back to mechanistic underpinnings (*e.g.*, recombination rate variation) determining the difference in relative importance of genetic drift and selection in different parts of the genome, and that a major proportion of the fixed differences we observe are the result of stochastic fixation of alternative alleles in the two species in allopatry. However, the observation that a larger proportion of autosomal fixed differences are present in exonic sequence or adjacent to genes indicates that at least a proportion of the fixed differences on the autosomes result from selection driving alternative alleles to fixation in *abietinus* and *tristis*, respectively. In addition, it is possible that stochastic allele frequency changes and random accumulation of fixed differences may lead to diverging phenotypes between species in allopatry, and it is therefore of interest to investigate the functional relevance of observed differences between species, even if it is apparent that the major fraction of these were not initially driven to fixation by adaptation to different environments (natural selection and ecological speciation) or by different preferences for secondary sexual traits (sexual selection). In the following paragraph, we discuss the detailed analyses of functions of genes in highly differentiated regions, and genes with alternative alleles fixed between the two species.

The common and the Siberian chiffchaff differ in morphology, plumage color, song, and migration patterns—traits that most likely are of importance for species recognition and lineage specific adaptations (Marova *et al.* 2017; Marova and Alekseev 2008; Marova and Leonovich 1993; Marova *et al.* 2013; Shipilina *et al.* 2017). Gene-flow between the species has been shown to lead to intermediate or mixed traits in hybrids and back-crosses (Marova *et al.* 2017; Marova and Alekseev 2008; Shipilina *et al.* 2017), and characterization of the genetic basis of these traits is of importance to understand species integrity and the evolutionary effects of introgression. We conducted several analyses to detect genes that might be of interest for understanding phenotypic differences between *abietinus* and *tristis* that may reflect species specific adaptations. First, we selected all genes that were located in genomic regions enriched for relative differentiation outliers, and analyzed if any functional categories were overrepresented in this class of genes. This analysis showed no significant enrichment in the allopatric comparison and a single term (regulation of cellular organization) in sympatry. Such an analysis is characteristically rough, and, although it may give some preliminary insights into gene categories associated with high differentiation, it is difficult to draw detailed conclusions regrading functional relevance (Vijay *et al.* 2016). To get more detailed information about genes in highly differentiated regions, we also selected the windows with the highest level of differentiation (*F_ST_*) within each of the regions enriched for relative differentiation (*F_ST_^Z^*) outliers, and found that these top windows overlapped with 26 protein coding genes. The genes were mainly involved in functions related to ion transport, expression regulation (transcription factors, histone binding, and phosphorylation), and muscle activity/development, but also associated with processes directly relevant to phenotypic traits of interest in our study system; for example, sensory perception of sound (*OTOG*), and pigmentation (*HPS5*) (Table 2). Both *OTOG* and *HPS5* are located in the peak of the differentiation island on chromosome 5. *OTOG* has been associated with sound perception and locomotion in mouse (*Mus musculus domesticus*) (Simmler *et al.* 2000), and otolith function (hearing and balance organ development) in zebra fish (*Danio rerio*) (Stooke-Vaughan *et al.* 2015), indicating a conserved function in hearing and balance organs across very divergent lineages. Song recognition is a key mechanism for mate choice in birds (Nowicki and Searcy 2005), and recent data indicate that allopatric *abietinus* and *tristis* only react to conspecific song (*e.g.*, Marova *et al.* 2017). It is therefore tempting to contemplate that genes involved in sensory perception of sound may play an important role for driving differences in song preference, and song learning processes, in vocally differentiated bird species like *abietinus* and *tristis*. *HPS5* is involved in biogenesis of organelles, and has been associated with melanocyte formation and pigmentation in zebra fish (Daly *et al.* 2013). Plumage pigmentation is also a key trait for mate recognition and speciation in birds (Price 2008), and, again, it is enticing to speculate around a potential role for this gene underlying specific plumage patterning in chiffchaff. As mentioned before, both *OTOG* and *HPS5* are found in the peak of the differentiation island on chromosome 5. If selection has played any part in the high allele frequency difference between *abietinus* and *tristis* in this region, one cannot rule out that genetic hitchhiking has resulted in reduced nucleotide diversity both in the selected region and in linked genes. Therefore, verification experiments will be needed to get detailed information about the phenotypic effects of variation within these genes.

Finally, we also analyzed all fixed differences observed between allopatric *abietinus* and *tristis* and selected the positions where the substitutions resulted in amino acid and splice signaling changes. Again, we found that several genes were related to metabolism and gene expression (transcription factors, RNA processing, and methylation), but there was a diverse array of other functions in this class of genes. Of particular interest were two genes that had multiple (*n* = 2) fixed differences resulting in nonsynonymous changes; *FYB* and *PRUNE2*. *FYB* has been shown to be involved in the innate immune response by stimulating interleukin-2 expression in T-cells in the mouse (Krause *et al.* 2000), while *PRUNE2* has been associated with many different functions (http://www.genecards.org/cgi-bin/carddisp.pl?gene=PRUNE2); the gene is, for example, associated with pyrophosphatase activity that could indicate involvement in lipid metabolism. Two additional genes contained both a nonsynonymous and a splice-altering fixed difference; *KDM4C* and *KIF24*. *KDM4C* regulates gene expression by targeting trimethylated histones in humans (Hillringhaus *et al.* 2011), and *KIF24* is a kinesin that is a microtubule remodelling ATPase likely involved in intracellular transport (Kobayashi *et al.* 2011). From these analyses combined, we hypothesize that some of the genes located in highly differentiated regions, or containing fixed differences at nonsynonymous and splice-altering sites, might be relevant to the observed morphological, ecological, and behavioral differences between *abietinus* and *tristis*. However, detailed additional studies are obviously needed before any of these can be considered strong candidates for relevant trait differences. Further ahead still is the identification of potential links between candidate genes for trait variation and reproductive isolating mechanisms, and it is unlikely that standard genome scan approaches can be used to trace such links (Baird 2017; Buerkle 2017; Ellegren and Wolf 2017; Elmer 2017; Feder *et al.* 2017; Jiggins and Martin 2017; Ravinet *et al.* 2017; Wagner and Mandeville 2017; Westram and Ravinet 2017). We foresee that the next step will be more extensive sampling within the sympatric region, including birds with intermediate phenotypes and using detailed morphological and behavioral studies, combined with resequencing efforts of candidate regions, to find significant associations between specific alleles and phenotypic traits of interest.

It should be noted that a reference-assisted assembly cannot detect structural changes between the target organism and the reference. A consequence might be that visual inspection of patterns of genetic variation might be affected. Since birds in general have conserved karyotypes and comparatively few large structural rearrangements (Ellegren 2009), this is likely not a major concern, especially when visual inspection is combined with analyses that do not depend on genomic location. As mentioned above, patterns of genetic diversity and differentiation in chiffchaff are remarkably consistent with observations in other avian taxa, but a detailed inspection of the genome-wide patterns reveals that a few rearrangements probably explain some observed abrupt changes in the patterns of genetic variation along chiffchaff chromosomes. This concerns inversions ranging from 4 to 25 Mb in chromosomes 3, 4, 5, 20, and Z (Figure S8 in File S1). The structural changes do not affect the qualitative interpretation of the results in this study since they constitute but a minor part of the genome, and do not overlap with any of the major differentiation islands observed.

To conclude, this study contributes to the increasing body of knowledge about genome divergence processes under natural settings, and highlights the chiffchaff species complex as an important model system to study the genomics of speciation and adaptation in songbirds. In a general perspective, this work will help in the overall understanding of the speciation process, and, in particular, how secondary contact affects patterns of genome differentiation between diverging lineages.

## Supplementary Material

Supplemental material is available online at www.g3journal.org/lookup/suppl/doi:10.1534/g3.117.300152/-/DC1.

## References

[bib1] Abbott R. J. Barton N. H. Good J. M., 2016 Genomics of hybridization and its evolutionary consequences. Mol. Ecol. 25: 2325–2332. 27145128 10.1111/mec.13685

[bib2] Arvidsson B. L. Neergaard R., 1991 Mate choice in the willow warbler—a field experiment. Behav. Ecol. Sociobiol. 29: 225–229.

[bib3] Backström N. Forstmeier W. Schielzeth H. Mellenius H. Nam K., 2010 The recombination landscape of the zebra finch *Taeniopygia guttata* genome. Genome Res. 20: 485–495. 20357052 10.1101/gr.101410.109 PMC2847751

[bib4] Baird S. J. E., 2017 The impact of high-throughput sequencing technology on speciation research: maintaining perspective. J. Evol. Biol. 30: 1482–1487. 28786190 10.1111/jeb.13099

[bib5] Barton N. H. Hewitt G. M., 1989 Adaptation, speciation and hybrid zones. Nature 341: 497–503. 2677747 10.1038/341497a0

[bib6] Bateson W., 1909 Heredity and variation in modern lights, pp. 85–101 in Darwin and Modern Science, edited by Seward A. C. Cambridge University Press, Cambridge, UK.

[bib8] Bernatchez L. Renaut S. Whiteley A. R. Derome N. Jeukens J., 2010 On the origin of species: insights from the ecological genomics of whitefish. Phil. Trans. Roy. Soc. B: Biol. 365: 1783–1800. 10.1098/rstb.2009.0274 PMC2871888 20439281

[bib9] Bernt M. Donath A. Juhling F. Externbrink F. Florentz C., 2013 MITOS: improved *de novo* metazoan mitochondrial genome annotation. Mol. Phyl. Evol. 69: 313–319. 10.1016/j.ympev.2012.08.023 22982435

[bib10] Buckling A. Maclean R. C. Brockhurst M. A. Colegrave N., 2009 The Beagle in a bottle. Nature 457: 824–829. 19212400 10.1038/nature07892

[bib11] Buerkle C. A., 2017 Inconvenient truths in population and speciation genetics point towards a future beyond allele frequencies. J. Evol. Biol. 30: 1498–1500. 28786192 10.1111/jeb.13106

[bib12] Burri R., 2017 a Dissecting differentiation landscapes: a linked selection’s perspective. J. Evol. Biol. 30: 1501–1505. 28786187 10.1111/jeb.13108

[bib13] Burri R., 2017 b Linked selection, demography and the evolution of correlated genomic landscapes in birds and beyond. Mol. Ecol. 26: 3853–3856. 28749613 10.1111/mec.14167

[bib14] Burri R. Nater A. Kawakami T. Mugal C. F. Olason P. I., 2015 Linked selection and recombination rate variation drive the evolution of the genomic landscape of differentiation across the speciation continuum of *Ficedula* flycatchers. Genome Res. 25: 1656–1665. 26355005 10.1101/gr.196485.115 PMC4617962

[bib15] Charlesworth B. Nordborg M. Charlesworth D., 1997 The effects of local selection, balanced polymorphism and background selection on equilibrium patterns of genetic diversity in subdivided populations. Genet. Res. 70: 155–174. 9449192 10.1017/s0016672397002954

[bib16] Chung H. Loehlin D. W. Dufour H. D. Vacarro K. Millar J. G., 2014 A single gene affects both ecological divergence and mate choice in *Drosophila*. Science 343: 1148–1151. 24526311 10.1126/science.1249998

[bib17] Cingolani P. Platts A. Wang L. L. Coon M. Nguyen T., 2012 A program for annotating and predicting the effects of single nucleotide polymorphisms, SnpEff. Fly (Austin) 6: 80–92. 22728672 10.4161/fly.19695 PMC3679285

[bib18] Claramunt S. Cracraft J., 2015 A new time tree reveals earth history’s imprint on the evolution of modern birds. Sci. Adv. 1: e1501005. 26824065 10.1126/sciadv.1501005 PMC4730849

[bib19] Coyne J. A. Orr H. A., 2004 Speciation. Sinauer Associates, Sunderland, MA.

[bib20] Cruickshank T. E. Hahn M. W., 2014 Reanalysis suggests that genomic islands of speciation are due to reduced diversity, not reduced gene flow. Mol. Ecol. 23: 3133–3157. 24845075 10.1111/mec.12796

[bib21] Daly C. M. Willer J. Gregg R. Gross J. M., 2013 *Snow white*, a zebrafish model of Hermansky-Pudlak Syndrome type 5. Genetics 195: 481–494. 23893484 10.1534/genetics.113.154898 PMC3781975

[bib22] Danecek P. Auton A. Abecasis G. Albers C. A. Banks E., 2011 The variant call format and VCFtools. Bioinfo. 27: 2156–2158. 10.1093/bioinformatics/btr330 PMC3137218 21653522

[bib23] del Hoyo J. Collar N., 2017 Siberian chiffchaff (*Phylloscopus tristis*), in Handbook of the Birds of the World Alive, edited by del Hoyo J. Elliott A. Sargatal J. Christie D. A. de Juana E. Lynx Edicions, Barcelona, Spain.

[bib24] del Hoyo J. Elliot A. Christie D. A., 2006 Handbook of the Birds of the World. Old World Flycatchers to Old World Warblers. Lynx Edicions, Barcelona, Spain.

[bib25] Dobzhansky T., 1940 Speciation as a stage in evolutionary divergence. Am. Nat. 74: 312–321.

[bib26] Dutoit L. Burri R. Nater A. Mugal C. F. Ellegren H., 2017 Genomic distribution and estimation of nucleotide diversity in natural populations: perspectives from the collared flycatcher (*Ficedula albicollis*) genome. Moil. Ecol. Res. 17: 586–597. 10.1111/1755-0998.12602 27717155

[bib27] Egan S. P. Ragland G. J. Assour L. Powell T. H. Hood G. R., 2015 Experimental evidence of genome-wide impact of ecological selection during early stages of speciation-with-gene-flow. Ecol. Lett. 18: 817–825. 26077935 10.1111/ele.12460 PMC4744793

[bib28] Ellegren H., 2009 Evolutionary stasis: the stable chromosomes of birds. Trends Ecol. Evol. 25: 283–291. 10.1016/j.tree.2009.12.004 20363047

[bib29] Ellegren H. Fridolfsson A. K., 1997 Male-driven evolution of DNA sequences in birds. Nat. Genet. 17: 182–184. 9326938 10.1038/ng1097-182

[bib30] Ellegren H. Parsch J., 2007 The evolution of sex-biased genes and sex-biased gene expression. Nat. Rev. Genet. 8: 689–698. 17680007 10.1038/nrg2167

[bib31] Ellegren H. Wolf J. B. W., 2017 Parallelism in genomic landscapes of differentiation, conserved genomic features and the role of linked selection. J. Evol. Biol. 30: 1516–1518. 28786191 10.1111/jeb.13113

[bib32] Ellegren H. Smeds L. Burri R. Olason P. Backström N., 2012 The genomics of species differentiation in *Ficedula* flycatchers. Nature 491: 756–760. 23103876 10.1038/nature11584

[bib33] Elmer K. R., 2017 Barrier loci and progress towards evolutionary generalities. J. Evol. Biol. 30: 1491–1493. 28786186 10.1111/jeb.13104

[bib34] Feder J. L. Nosil P., 2010 The efficacy of divergence hitch-hiking in generating genomic islands during ecological speciation. Evolution 64: 1724–1747. 10.1111/j.1558-5646.2010.00943.x 20624183

[bib35] Feder J. L. Egan P. S. Nosil P., 2012 The genomics of speciation-with-gene-flow. Trends Genet. 28: 342–350. 22520730 10.1016/j.tig.2012.03.009

[bib36] Feder J. L. Flaxman S. M. Egan S. P. Comeault A. A. Nosil P., 2013 Geographic mode of speciation and genomic divergence. Annu. Rev. Ecol. Evol. Syst. 44: 73–97.

[bib37] Feder J. L. Nosil P. Gompert Z. Flaxman S. M. Schilling M. P., 2017 Barnacles, barrier loci and the systematic building of species. J. Evol. Biol. 30: 1494–1497. 28786183 10.1111/jeb.13105

[bib38] Ferchaud A. L. Hansen M. M., 2016 The impact of selection, gene flow and demographic history on heterogeneous genomic divergence: three-spine sticklebacks in divergent environments. Mol. Ecol. 25: 238–259. 26412233 10.1111/mec.13399

[bib39] Frichot E., 2014 Fast and efficient estimation of individual ancestry coefficients. Genetics 196: 973–983. 24496008 10.1534/genetics.113.160572 PMC3982712

[bib40] Frichot E., 2015 LEA: an R package for landscape and ecological association studies. Methods Ecol. Evol. 6: 925–929.

[bib41] Gompert Z. Comeault A. A. Farkas T. E. Feder J. L. Parchman T. L., 2014 Experimental evidence for ecological selection on genome variation in the wild. Ecol. Lett. 17: 369–379. 24354456 10.1111/ele.12238 PMC4261992

[bib42] Gurevich A. Saveliev V. Vyahhi N. Tesler G., 2013 QUAST: quality assessment tool for genome assemblies. Bioinfo. 29: 1072–1075. 10.1093/bioinformatics/btt086 PMC3624806 23422339

[bib43] Hahn C., 2013 Reconstructing mitochondrial genomes directly from genomic next-generation sequencing reads—a baiting and iterative mapping approach. Nucleic Acids Res. 41: e129. 23661685 10.1093/nar/gkt371 PMC3711436

[bib44] Harr B., 2006 Genomic islands of differentiation between house mouse subspecies. Genome Res. 16: 730–737. 16687734 10.1101/gr.5045006 PMC1473184

[bib45] Helbig A. J. Martens J. Seibold I. Henning F. Schottler B., 1996 Phylogeny and species limits in the Palaearctic chiffchaff *Phylloscopus collybita* complex: mitochondrial genetic differentiation and bioacoustic evidence. Ibis 138: 650–666.

[bib46] Hillringhaus L. Yue W. W. Rose N. R. Ng S. S. Gileadi C., 2011 Structural and evolutionary basis for the dual substrate selectivity of human KDM4 histone demethylase family. J. Biol. Chem. 286: 41616–41625. 21914792 10.1074/jbc.M111.283689 PMC3308871

[bib47] Hurst L. D. Ellegren H., 1998 Sex biases in the mutation rate. Trends Genet. 14: 446–452. 9825672 10.1016/s0168-9525(98)01577-7

[bib48] Irwin D. E. Alcaide M. Delmore K. E. Irwin J. H. Owens G. L., 2016 Recurrent selection explains parallel evolution of genomic regions of high relative but low absolute differentiation in a ring species. Mol. Ecol. 25: 4488–4507. 27484941 10.1111/mec.13792

[bib49] Jiggins C. D. Martin S. H., 2017 Glittering gold and the quest for Isla de Muerta. J. Evol. Biol. 30: 1509–1511. 28786188 10.1111/jeb.13110

[bib50] Jiggins C. D. McMillan W. O. Neukirchen W. Mallet J., 1996 What can hybrid zones tell us about speciation? The case of *Heliconius erato* and *H. himera* (Lepidoptera: Nymphalidae). Biol. J. Linn. Soc. Lond. 59: 221–242.

[bib51] Jones F. C. Grabherr M. G. Chan Y. F. Russell P. Mauceli E., 2012 The genomic basis of adaptive evolution in threespine sticklebacks. Nature 484: 55–61. 22481358 10.1038/nature10944 PMC3322419

[bib52] Joron M. Papa R. Beltran M. Chamberlain N. Mavarez J., 2006 A conserved supergene locus controls colour pattern diversity in *Heliconius* butterflies. PLoS Biol. 4: e303. 17002517 10.1371/journal.pbio.0040303 PMC1570757

[bib53] Katoh K. Standley D. M., 2013 MAFFT multiple sequence alignment software version 7: improvements in performance and usability. Mol. Biol. Evol. 30: 772–780. 23329690 10.1093/molbev/mst010 PMC3603318

[bib54] Kawakami T. Smeds L. Backström N. Husby A. Qvarnström A., 2014 A high-density linkage map enables a second-generation collared flycatcher genome assembly and reveals the patterns of avian recombination rate variation and chromosomal evolution. Mol. Ecol. 23: 4035–4058. 24863701 10.1111/mec.12810 PMC4149781

[bib55] Kobayashi T. Tsang W. Y. Li J. Lane W. Dynlacht B. D., 2011 Centriolar kinesin Kif24 interacts with CP110 to remodel microtubules and regulate ciliogenesis. Cell 145: 914–925. 21620453 10.1016/j.cell.2011.04.028

[bib56] Komarova A. F. Shipilina D. A., 2010 Hybrid population of eastern European and Siberian forms of chiffchaff (*Phylloscopus collybita abietinus*, Ph. (c.) tristis) in the Arkhangelsk area., pp. 211–212 in *XVII International Conference of Students*, *Post-Graduate Students and Young Scientists* MAKS Press, Lomonosov.

[bib57] Korneliussen T. S. Albrechtsen A. Nielsen R., 2014 ANGSD: analysis of next generation sequencing data. Bioinfo. 15: 356. 10.1186/s12859-014-0356-4 PMC4248462 25420514

[bib58] Krause M. Sechi A. S. Konradt M. Monner D. Gertler F. B., 2000 Fyn-binding protein (Fyb):SLP-76–associated protein (SLAP), Ena: vasodilator-stimulated phosphoprotein (VASP) proteins and the Arp2: 3 complex link T cell receptor (TCR) signaling to the actin cytoskeleton. J. Cell Biol. 149: 181–194. 10747096 10.1083/jcb.149.1.181 PMC2175102

[bib59] Kumar S. Stecher G. Tamura K., 2016 MEGA7: molecular evolutionary genetics analysis version 7.0 for bigger datasets. Mol. Biol. Evol. 33: 1870–1874. 27004904 10.1093/molbev/msw054 PMC8210823

[bib60] Lampa S. Dahlö M. Olason P. I. Hagberg J. Spjuth O., 2013 Lessons learned from implementing a national infrastructure in Sweden for storage and analysis of next-generation sequencing data. Gigasci. 25: 9. 10.1186/2047-217X-2-9 PMC3704847 23800020

[bib61] Li H. Durbin R., 2010 Fast and accurate long-read alignment with Burrows-Wheeler transformation. Bioinfo. 26: 589–595. 10.1093/bioinformatics/btp698 PMC2828108 20080505

[bib62] Li H. Handsaker B. Wysoker A. Fennell T. Ruan J., 2009 The sequence alignment/map format and SAMtools. Bioinfo. 25: 2078–2079. 10.1093/bioinformatics/btp352 PMC2723002 19505943

[bib63] Lindholm A., 2008 Mixed songs of chiffchaffs in northern Russia. Alula 3: 108–115.

[bib64] Lohse K., 2017 Come on feel the noise—from metaphors to null models. J. Evol. Biol. 30: 1506–1508. 28786185 10.1111/jeb.13109

[bib65] Lyu N. Li J. Sun Y.-H., 2016 Can simple songs express useful signals for mate choice? Avian Res. 7: e10.

[bib66] Mank J. E., 2009 Sex chromosomes and the evolution of sexual dimorphism: lessons from the genome. Am. Nat. 173: 141–150. 20374139 10.1086/595754

[bib67] Mank J. E. Axelsson E. Ellegren H., 2007 Fast-X on the Z: rapid evolution of sex-linked genes in birds. Genome Res. 17: 618–624. 17416747 10.1101/gr.6031907 PMC1855182

[bib68] Mank J. E. Vicoso B. Berlin S. Charlesworth B., 2010 Effective population size and the faster-X effect: empirical results and their interpretation. Evolution 64: 663–674. 19796145 10.1111/j.1558-5646.2009.00853.x

[bib69] Marova I. Shipilina D. Fedorov V. V. Alekseev V. Ivanitskii V., 2017 Interaction between common and Siberian chiffchaff in a contact zone. Ornis Fenn. 94: 66–81.

[bib70] Marova I. M. Alekseev V. N., 2008 Population structure and distribution of vocal dialects of chiffchaff (*Phylloscopus collybita*) in southern Ural mountains. Proceedings of the South Ural State Natural Reserve, Vol. 1, pp. 306–318.

[bib71] Marova I. M. Leonovich V. V., 1993 Hybridization between Siberian (*Phylloscopus collybita tristis*) and east European (*Ph. collybita abietinus*) chiffchaffs in the area of sympatry. Sbornik Trudov Zoologicheskogo Muzeya. Moskovskogo Gosudarstvennogo Universiteta 30: 147–163 (in Russian).

[bib72] Marova I. M. Fedorov V. V. Shipilina D. A. Alekseev V. N., 2009 Genetic and vocal differentiation in hybrid zones of passerine birds: Siberian and European chiffchaffs (*Phylloscopus [collybita] tristis* and *Ph. [c.] abietinus*) in the southern Urals. Dokl. Biol. Sci. 427: 384–386. 19760890 10.1134/s0012496609040231

[bib73] Marova I. M. Shipilina D. A. Fedorov V. V. Ivanitskii V. V., 2013 Siberian and East European chiffchaffs: geographical distribution, morphological features, vocalization, phenomenon of mixed singing, and evidences of hybridization in sympatry zone in El Mosquitero Ibérico, edited by Rodríguez N. Garcia J. Copete J. L. Grupo Iberico de Anillamiento, Leon, Spain.

[bib74] Martens J. Meinche C., 1989 Der sibirische zilpzalp (*Phylloscopus collybita tristis*): gesang und reaction einer mitteleuropäischen population im freilandversuch. J. Ornithol. 130: 455–473.

[bib75] Martin S. H. Dasmahapatra K. K. Nadeau N. J. Salazar C. Walters J. R., 2013 Genome-wide evidence for speciation with gene flow in *Heliconius* butterflies. Genome Res. 23: 1817–1828. 24045163 10.1101/gr.159426.113 PMC3814882

[bib76] McKenna A. Hanna M. Banks E. Sivachenko A. Cibulskis K., 2010 The genome analysis toolkit: a MapReduce framework for analyzing next-generation DNA sequencing data. Genome Res. 20: 1297–1303. 20644199 10.1101/gr.107524.110 PMC2928508

[bib77] Muller H. J., 1942 Isolating mechanisms, evolution and temperature. Biol. Symp. 6: 71–125.

[bib78] Nabholz B. Lanfear R. Fuchs J., 2016 Body mass-corrected molecular rate for bird mitochondrial DNA. Mol. Ecol. 25: 4438–4449. 27483387 10.1111/mec.13780

[bib79] Nachman M. W. Payseur B. A., 2012 Recombination rate variation and speciation: theoretical predictions and empirical results from rabbits and mice. Phil. Trans. Roy. Soc. B: Biol. 367: 409–421. 10.1098/rstb.2011.0249 PMC3233716 22201170

[bib80] Noor M. A. F. Bennett S. M., 2009 Islands of speciation or mirages in the desert? Examining the role of restricted recombination in maintaining species. Heredity 103: 439–444. 19920849 10.1038/hdy.2009.151 PMC2809014

[bib81] Noor M. A. F. Feder J. L., 2006 Speciation genetics: evolving approaches. Nat. Rev. Genet. 7: 851–861. 17033626 10.1038/nrg1968

[bib82] Nosil P. Feder J. L., 2012 Genomic divergence during speciation: causes and consequences. Phil. Trans. Roy. Soc. B: Biol. 367: 332–342. 10.1098/rstb.2011.0263 PMC3233720 22201163

[bib83] Nowicki S. Searcy W. A., 2005 Song and mate choice in birds: how the development of behavior helps us understand function. Auk 122: 1–14.

[bib84] Okonechnikov K. Conesa A. García-Alcalde F., 2015 Qualimap 2: advanced multi-sample quality control for high-throughput sequencing data. Bioinfo. 32: 292–294. 10.1093/bioinformatics/btv566 PMC4708105 26428292

[bib85] Oyler-McCance S. J. Cornman R. S. Jones K. L. Fike J. A., 2015 Z chromosome divergence, polymorphism and relative effective population size in a genus of lekking birds. Heredity 115: 452–459. 26014526 10.1038/hdy.2015.46 PMC4611240

[bib86] Payseur B. A. Rieseberg L. H., 2016 A genomic perspective on hybridization and speciation. Mol. Ecol. 25: 2337–2360. 26836441 10.1111/mec.13557 PMC4915564

[bib87] Pennisi E., 2014 Disputed islands. Science 345: 611–613. 25104366 10.1126/science.345.6197.611

[bib88] Poelstra J. W. Vijay N. Bossu C. M. Lantz H. Ryll B., 2014 The genomic landscape underlying phenotypic integrity in the face of gene flow in crows. Science 344: 1410–1414. 24948738 10.1126/science.1253226

[bib89] Presgraves D. C., 2008 Sex-chromosomes and speciation in *Drosophila*. Trends Genet. 24: 336–343. 18514967 10.1016/j.tig.2008.04.007 PMC2819171

[bib90] Price T. D., 2008 Speciation in Birds. Roberts & Company Publishers, Greenwood Village, CO.

[bib91] Purcell S. Neale B. Todd-Brown K. Thomas L. Ferreira M. A. R., 2007 PLINK: a tool set for whole-genome association and population-based linkage analyses. Am. J. Hum. Genet. 81: 559–575. 17701901 10.1086/519795 PMC1950838

[bib92] Quinlan A. R. Hall I. M., 2010 BEDTools: a flexible suite of utilities for comparing genomic features. Bioinfo. 26: 841–842. 10.1093/bioinformatics/btq033 PMC2832824 20110278

[bib93] Qvarnström A. Bailey R. I., 2009 Speciation through evolution of sex-linked genes. Heredity 102: 4–15. 18781167 10.1038/hdy.2008.93

[bib94] Qvarnström A. Rice A. M. Ellegren H., 2010 Speciation in *Ficedula* flycatchers. Phil. Trans. Roy. Soc. B: Biol. 365: 1841–1852. 10.1098/rstb.2009.0306 PMC2871891 20439285

[bib95] Ravinet M. Faria R. Butlin R. K. Galindo J. Bierne N., 2017 Interpreting the genomic landscape of speciation: a road map for finding barriers to gene flow. J. Evol. Biol. 30: 1450–1477. 28786193 10.1111/jeb.13047

[bib96] Renaut S. Grassa C. J. Yeaman S. Moyers B. T. Lai Z., 2013 Genomic islands of divergence are not affected by geography of speciation in sunflowers. Nat. Commun. 4: 1827. 23652015 10.1038/ncomms2833

[bib97] Rogers S. M. Bernatchez L., 2007 The genetic architecture of ecological speciation and the association with signature of selection in natural lake whitefish (*Coregonus sp. Salmonidae*) species pairs. Mol. Biol. Evol. 24: 1423–1428. 17404398 10.1093/molbev/msm066

[bib98] Sackton T. B. Corbett-Detig R. B. Nagaraju J. Vaishna L. Arunkumar K. P., 2014 Positive selection drives faster-Z evolution in silkmoths. Evolution 68: 2331–2342. 24826901 10.1111/evo.12449 PMC4122604

[bib99] Sætre G. P. Sæther S. A., 2010 Ecology and genetics of speciation in *Ficedula* flycatchers. Mol. Ecol. 19: 1091–1106. 20163542 10.1111/j.1365-294X.2010.04568.x

[bib100] Sætre G.-P. Moum T. Bures S. Král M. Adamjan M., 1997 A sexually selected character displacement in flycatchers reinforces premating isolation. Nature 387: 589–592.

[bib101] Saitou N. Nei M., 1987 The neighbor-joining method: a new method for reconstructing phylogenetic trees. Mol. Biol. Evol. 4: 406–425. 3447015 10.1093/oxfordjournals.molbev.a040454

[bib102] Sambrook J. Fritsch E. F. Maniatis T., 1989 Molecular Cloning: A Laboratory Manual. Cold Spring Harbor Laboratory Press, Cold Spring Harbor, NY.

[bib103] Schluter D., 2009 Evidence for ecological speciation and its alternative. Science 323: 737–741. 19197053 10.1126/science.1160006

[bib104] Seehausen O. Butlin R. K. Keller I. Wagner C. E. Boughman J. W., 2014 Genomics and the origin of species. Nat. Rev. Genet. 15: 176–192. 24535286 10.1038/nrg3644

[bib105] Selvatti A. P. Gonzaga L. P. Russo C. A., 2015 A Paleogene origin for crown passerines and the diversification of the oscines in the new world. Mol. Phyl. Evol. 88: 1–15. 10.1016/j.ympev.2015.03.018 25837731

[bib106] Shipilina D. Serbyn M. Ivanitskii V. Marova I. Backström N., 2017 Patterns of genetic, phenotypic, and acoustic variation across a chiffchaff (*Phylloscopus collybita abietinus/tristis*) hybrid zone. Ecol. Evol. 7: 2169–2180. 28405281 10.1002/ece3.2782 PMC5383471

[bib107] Simao F. A. Waterhouse R. M. Ioannidis P. Kriventseva E. V. Zdobnov E. M., 2015 BUSCO: assessing genome assembly and annotation completeness with single-copy orthologs. Bioinfo. 31: 3210–3212. 10.1093/bioinformatics/btv351 26059717

[bib108] Simmler M.-C. Cohen-Salmon M. El-Amraoui A. Guillaud L. Benichou J.-C., 2000 Targeted disruption of *OTOG* results in deafness and severe imbalance. Nat. Genet. 24: 139–143. 10655058 10.1038/72793

[bib109] Singhal S. Leffler E. M. Sannareddy K. Turner I. Venn O., 2015 Stable recombination hotspots in birds. Science 350: 928–932. 26586757 10.1126/science.aad0843 PMC4864528

[bib110] Smit, A., R. Hubley, and P. Green, 2013–2015 RepeatMasker open-4.0. Available at: http://www.repeatmasker.org/.

[bib111] Stepanyan L. S., 1990 Synopsis of the Ornithological Fauna of the Soviet Union. Nauka, Moscow.

[bib112] Stooke-Vaughan G. A. Obholzer N. D. Baxendale S. Megason S. G. Whitfield T. T., 2015 Otolith tethering in the zebrafish otic vesicle requires Otogelin and alpha-Tectorin. Development 142: 1137–1145. 25758224 10.1242/dev.116632 PMC4360185

[bib113] Storz J. F., 2005 Using genome scans of DNA polymorphism to infer adaptive population divergence. Mol. Ecol. 14: 671–688. 15723660 10.1111/j.1365-294X.2005.02437.x

[bib114] Svensson L., 1992 Identification Guide to European Passerines. Cornell University, British Trust for Ornithology, Ithaca, NY.

[bib115] Tamura K. Nei M. Kumar S., 2004 Prospects for inferring very large phylogenies by using the neighbor-joining method. Proc. Natl. Acad. Sci. USA 101: 11030–11035. 15258291 10.1073/pnas.0404206101 PMC491989

[bib116] Ticehurst C., 1938 A Systematic Review of the Genus Phylloscopus. Oxford University Press, London.

[bib117] Turelli M. Orr H. A., 2000 Dominance, epistasis and the genetics of postzygotic isolation. Genetics 154: 1663–1679. 10747061 10.1093/genetics/154.4.1663 PMC1461023

[bib118] Turner T. L. Hahn M. W. Nuzhdin S. V., 2005 Genomic islands of speciation in *Anopheles gambiae*. PLoS Biol. 3: 1572–1578. 10.1371/journal.pbio.0030285 PMC1182689 16076241

[bib119] Van den Berg A. B., 2009 Calls, identification and taxonomy of sSiberian chiffchaff: an analysis. Dutch Birding 31: 79–85.

[bib120] Van Doren B. M. Campagna L. Helm B. Illera J. C. Lovette I. J., 2017 Correlated patterns of genetic diversity and differentiation across an avian family. Mol. Ecol. 15: 3982–3997. 10.1111/mec.14083 28256062

[bib121] Via S., 2009 Natural selection in action during speciation. Proc. Natl. Acad. Sci. USA 106: 9939–9946. 19528641 10.1073/pnas.0901397106 PMC2702801

[bib122] Via S. West J., 2008 The genetic mosaic suggests a new role for hitchhiking in ecological speciation. Mol. Ecol. 17: 4334–4345. 18986504 10.1111/j.1365-294X.2008.03921.x

[bib123] Vicoso B. Charlesworth B., 2006 Evolution on the X chromosome: unusual patterns and processes. Nat. Rev. Genet. 7: 645–653. 16847464 10.1038/nrg1914

[bib124] Vicoso B. Charlesworth B., 2009 Effective population size and the faster-X effect: an extended model. Evolution 63: 2413–2426. 19473388 10.1111/j.1558-5646.2009.00719.x

[bib125] Vijay N. Bossu C. M. Poelstra J. W. Weissensteiner M. H. Suh A., 2016 Evolution of heterogeneous genome differentiation across multiple contact zones in a crow species complex. Nat. Commun. 7: 13195. 27796282 10.1038/ncomms13195 PMC5095515

[bib126] Vijay N. Weissensteiner M. Burri R. Kawakami T. Ellegren H., 2017 Genome-wide patterns of variation in genetic diversity are shared among populations, species and higher order taxa. Mol. Ecol. 26: 4284–4295. 28570015 10.1111/mec.14195

[bib127] Wagner C. E. Mandeville E. G., 2017 Speciation, species persistence and the goals of studying genomic barriers to gene flow. J. Evol. Biol. 30: 1512–1515. 28786196 10.1111/jeb.13112

[bib128] Westram A. M. Ravinet M., 2017 Land ahoy? Navigating the genomic landscape of speciation while avoiding shipwreck. J. Evol. Biol. 30: 1522–1525. 28786189 10.1111/jeb.13129

[bib129] Wilson Sayres M. A. Makova K. D., 2011 Genome analyses substantiate male mutation bias in many species. BioEssays 33: 938–945. 22006834 10.1002/bies.201100091 PMC4600401

[bib130] Wolf J. B. Ellegren H., 2017 Making sense of genomic islands of differentiation in light of speciation. Nat. Rev. Genet. 18: 87–100. 27840429 10.1038/nrg.2016.133

[bib131] Wolf J. B. W. Lindell J. Backström N., 2010 Speciation genetics: current status and evolving approaches. Phil. Trans. Roy. Soc. B: Biol. 365: 1717–1733. 10.1098/rstb.2010.0023 PMC2871893 20439277

[bib132] Wu C.-I. Ting C.-T., 2004 Genes and speciation. Nat. Rev. Genet. 5: 114–122. 14735122 10.1038/nrg1269

[bib133] Zhang G. Li C. Li Q. Li B. Larkin D. M., 2014 Comparative genomics reveals insights into avian genome evolution and adaptation. Science 346: 1311–1320. 25504712 10.1126/science.1251385 PMC4390078

[bib134] Zheng X., 2012 A high-performance computing toolset for relatedness and principal component analysis of SNP data. Bioinfo. 28: 3326–3328. 10.1093/bioinformatics/bts606 PMC3519454 23060615

