# Supplementary material for: Heterogeneous Patterns of Genetic Diversity and Differentiation in European and Siberian Chiffchaff (Phylloscopus collybita abietinus/P. tristis)
Source: G3 (Bethesda). 2017 Oct 20;7(12):3983–98. doi: 10.1534/g3.117.300152 (PMC5714495; doi:10.1534/g3.117.300152)

Supplementary Tables 1A and 1B

Summary of sequencing efforts and yield before and after quality filtering (Q-score ≥ 30). A) deep sequencing of 180 and 380 bp insert size, paired-end Illumina HiSeq (100 bp read length) libraries of *Pcol09* (*abietinus* allopatric) used for reference assisted genome assembly. B) re-sequencing of 10 allopatric *abietinus* (*Pcol01-Pcol12*), 10 allopatric *tristis* (*Pcol13-Pcol19 + Pcol510-Pcol704*), 10 sympatric *abietinus* (*Pcol24-Pcol35*), and 10 sympatric *tristis* (*Pcol20-Pcol23 + Pcol37-Pcol42*) with paired-end, 360 bp insert size Illumina HiSeq (100 bp read length) libraries. #reads = number of read pairs per library / individual, Acc. No. = individual accession numbers, Coverage = mean and variance in coverage across the assembly. Sympatric samples were collected in both the Northern (N) and the Southern (S) part of the region.

Supplementary Table 1A

Library	Ind.	# reads (*10^6)	Read	Acc. No.	# bp (Gb)	# filt. bp (Gb)	Coverage
180 bp	*Pcol09*	237.6	Read 1	ERS1811975	23.8	22.2	25.1 ± 140.5
1st run		237.6	Read 2		23.8	21.4	25.1 ± 140.5
180bp	*Pcol09*	235.1	Read 1	ERS1811976	23.5	22.0	25.1 ± 140.3
2nd run		235.1	Read2		23.5	21.1	25.1 ± 140.3
380 bp	*Pcol09*	18.4	Read 1	ERS1811977	1.8	1.7	2.1 ± 13.2
1st run		18.4	Read 2		1.8	1.6	2.1 ± 13.2
380bp	*Pcol09*	9.7	Read 1	ERS1811978	1.0	1.0	1.1 ± 4.8
2nd run		9.7	Read 2		1.0	0.9	1.1 ± 4.8
**Total**		**1,001.6**			**100.2**	**91.9**	**106.8**

Supplementary Table 1B

Population	Ind.	# reads	Acc. No.	# bp (Gb)	# filt. bp (Gb)	Coverage
abietinus, allopatry	*Pcol01*	18.6M	ERS1811076	3.8	3.6	3.9 ± 14.2
abietinus, allopatry	*Pcol02*	22.9M	ERS1811965	4.6	4.4	4.6 ± 15.9
abietinus, allopatry	*Pcol03*	20.2M	ERS1811973	4.0	3.8	4.1 ± 16.2
abietinus, allopatry	*Pcol04*	23.2M	ERS1811966	4.6	4.4	4.6 ± 18.7
abietinus, allopatry	*Pcol06*	20.3M	ERS1811967	4.0	3.8	4.1 ± 18.1
abietinus, allopatry	*Pcol07*	21.4M	ERS1811968	4.2	4.0	4.6 ± 17.6
abietinus, allopatry	*Pcol09*	18.4M	ERS1811969	3.6	3.6	3.9 ± 14.0
abietinus, allopatry	*Pcol10*	16.5M	ERS1811970	3.4	3.2	3.5 ± 14.8
abietinus, allopatry	*Pcol11*	13.8M	ERS1811971	2.8	2.6	2.7 ± 11.1
abietinus, allopatry	*Pcol12*	15.7M	ERS1811972	3.2	3.0	3.2 ± 13.8
tristis, allopatry	*Pcol13*	18.9M	ERS1815785	3.8	3.4	3.7 ± 31.6
tristis, allopatry	*Pcol14*	21.1M	ERS1815786	4.2	3.8	3.8 ± 42.1
tristis, allopatry	*Pcol15*	18.4M	ERS1815787	3.6	3.4	3.6 ± 35.9
tristis, allopatry	*Pcol16*	18.2M	ERS1815788	3.6	3.4	3.3 ± 36.0
tristis, allopatry	*Pcol17*	18.2M	ERS1815789	3.6	3.4	3.4 ± 33.0
tristis, allopatry	*Pcol18*	19.6M	ERS1815790	4.0	3.6	3.4 ± 28.1
tristis, allopatry	*Pcol19*	19.2M	ERS1815791	3.8	3.6	3.4 ± 30.0
tristis, allopatry	*Pcol510*	16.9M	ERS1815792	3.4	3.0	3.4 ± 16.8
tristis, allopatry	*Pcol703*	14.3M	ERS1815793	2.8	2.4	1.0 ± 34.9
tristis, allopatry	*Pcol704*	18.1M	ERS1815794	3.6	3.2	3.4 ± 19.5
tristis, sympatry (N)	*Pcol20*	15.2M	ERS1816475	3.0	3.0	2.9 ± 26.5
tristis, sympatry (N)	*Pcol21*	16.3M	ERS1816476	3.2	3.2	3.1 ± 30.9

tristis, sympatry (N)	*Pcol22*	13.4M	ERS1816477	2.6	2.6	2.5 ± 21.3
tristis, sympatry (N)	*Pcol23*	17.7M	ERS1816478	3.6	3.4	3.3 ± 25.2
abietinus, sympatry (N)	*Pcol24*	14.0M	ERS1816465	2.8	2.6	2.4 ± 21.3
abietinus, sympatry (N)	*Pcol25*	15.2M	ERS1816466	3.0	3.0	2.7 ± 27.5
abietinus, sympatry (N)	*Pcol26*	25.4M	ERS1816467	5.0	4.8	3.5 ± 28.8
abietinus, sympatry (N)	*Pcol27*	21.2M	ERS1816468	4.2	4.0	3.7 ± 32.7
abietinus, sympatry (N)	*Pcol29*	19.3M	ERS1816469	3.8	3.8	3.6 ± 26.3
abietinus, sympatry (N)	*Pcol31*	16.3M	ERS1816470	3.2	3.0	3.3 ± 17.9
abietinus, sympatry (S)	*Pcol32*	16.9M	ERS1816471	3.4	3.0	3.5 ± 15.3
abietinus, sympatry (S)	*Pcol33*	19.3M	ERS1816472	3.8	3.6	3.9 ± 24.4
abietinus, sympatry (S)	*Pcol34*	17.1M	ERS1816473	3.4	3.2	3.6 ± 14.1
abietinus, sympatry (S)	*Pcol35*	16.9M	ERS1816474	3.4	3.0	3.6 ± 16.3
tristis, sympatry (S)	*Pcol37*	19.2M	ERS1816479	3.8	3.6	3.9 ± 32.8
tristis, sympatry (S)	*Pcol38*	21.6M	ERS1816480	4.4	4.0	4.0 ± 50.0
tristis, sympatry (S)	*Pcol39*	17.8M	ERS1816481	3.6	3.2	3.1 ± 24.3
tristis, sympatry (S)	*Pcol41*	21.0M	ERS1816482	4.2	3.8	4.0 ± 33.0
tristis, sympatry (S)	*Pcol42*	18.7M	ERS1816483	3.8	3.4	3.7 ± 34.0
tristis, sympatry (S)	*Pcol47*	19.6M	ERS1816484	4.0	3.6	3.6 ± 43.2

Supplementary Table 2.

Assembly statistics for the reference assisted genome assembly of the common chiffchaff based on the fAlb15 version of the *Ficedula albicollis* genome assembly (Ellegren *et al.* 2012; Kawakami *et al.* 2014).

**Assembly statistics**	
Scaffold count	33
Total scaffold length	1.04 GB
Longest scaffold	157.6 MB
Scaffold N50 length	70.4 MB
Scaffold N90 length	14.9 MB

Supplementary Table 3.

Results from the coverage assessment of the chiffchaff genome assembly using BUSCO with eukaryote (Euk., n = 429 genes) and vertebrate (Vert., n = 2,586 genes) gene sets and analogous comparisons with the common crow (*Corvus*) (Poelstra *et al.* 2014; Vijay *et al.* 2016) and the collared flycatcher (*Ficedula*) (Ellegren *et al.* 2012; Kawakami *et al.* 2014) genome assemblies, respectively. Complete = completely covered genes, single-copy = completely covered single copy genes, duplicated = completely covered duplicated genes, fragmented = partly covered genes, missing = completely missing genes. The percentages of the total gene counts are given in brackets after the counts. Note that single-copy genes and duplicated genes are subsets of the complete genes.

**BUSCO gene set**	**Complete**	**Single-copy**	**Duplicated**	**Fragmented**	**Missing**
Chiffchaff Euk.	216 (50.3)	213 (49.7)	3 (0.7)	21 (4.9)	192 (44.8)
Chiffchaff Vert.	2,093 (80.9)	1,975 (76.4)	118 (4.6)	167 (6.5)	326 (12.6)
Ficedula Euk.	216 (50.3)	212 (49.4)	4 (0.9)	18 (4.2)	195 (45.5)
Ficedula Vert.	2,400 (92.8)	2,210 (85.5)	190 (7.3)	121 (4.7)	65 (2.5)
Corvus Euk.	216 (50.3)	212 (49.4)	4 (0.9)	28 (6.5)	185 (43.1)
Corvus Vert.	2,182 (84.4)	2,077 (80.3)	105 (4.1)	143 (5.5)	261 (10.1)

Supplementary Table 4.

Average levels of mtDNA genetic distances (substitutions per site) between population pairs as estimated with the Maximum Composite Likelihood (Tamura *et al.* 2004) and rate variation among sites modeled using a gamma distribution with shape parameter 1 as implemented in MEGA7 (Kumar *et al.* 2016). Ambiguous positions were removed for pairwise comparisons. Between species comparisons in allopatry and sympatry are indicated with italics font style.

	**Allopatric *abietinus***	**Allopatric *tristis***	**Sympatric *abietinus***
**Allopatric *abietinus***			
**Allopatric *tristis***	*0.0213 ± 0.0008*		
**Sympatric *abietinus***	0.0127 ± 0.0004	0.0120 ± 0.0005	
**Sympatric *tristis***	0.0209 ± 0.0004	0.0039 ± 0.0002	*0.0117 ± 0.0004*

Supplementary Table 5A.

Genomic locations of regions enriched (≥10 F_{ST}^{Z} outlier windows in block of 50 consecutive windows) for relative differentiation outliers in the comparison of allopatric *abietinus* and *tristis*.

Chromosome	Start position	End position
1	102540000	103540000
1	104390000	104400000
1A	47790000	48790000
1A	51400000	52400000
2	59400000	59900000
2	60440000	60940000
3	12610000	13610000
4	15880000	16380000
5	4730000	5230000
5	12580000	13580000
7	5210000	6210000
8	25050000	26050000
9	1520000	2520000
10	11690000	12690000
10	12900000	13900000
10	14630000	15130000
12	7880000	8880000
12	10300000	11800000
28	6120000	6620000

Supplementary Table 5B.

Genomic locations of regions enriched (≥10 F_{ST}^{Z} outlier windows in block of 50 consecutive windows) for relative differentiation outliers in the comparison of sympatric *abietinus* and *tristis*.

Chromosome	Start position	End position
1	103160000	103660000
1	103730000	105230000
1A	48810000	49310000
5	5180000	5680000
5	5700000	6200000
5	12700000	14700000
10	12000000	12500000
10	13000000	15000000
12	7640000	12140000
13	6410000	8410000
17	3370000	3870000
20	6210000	6220000
21	2000000	3000000

Supplementary Table 6A.

A list of protein coding genes located in the relative differentiation (F_{ST}^{Z}) outliers between allopatric (grey, n = 238) and sympatric (red, n = 274) *abietinus* and *tristis*. The orthologous *Ficedula albicollis* ensemble entry ID and the corresponding gene name is given (when available).

Gene ID	Gene name	Gene ID	Gene name
ENSFALG00000000780	*PIK3C2A*	ENSFALG00000000754	*KCNC1*
ENSFALG00000000784	*RPS13*	ENSFALG00000000758	*MYOD1*
ENSFALG00000000789	*PLEKHA7*	ENSFALG00000000765	*OTOG*
ENSFALG00000000793		ENSFALG00000000770	
ENSFALG00000000798	*SOX6*	ENSFALG00000000775	
ENSFALG00000001640	*NAV2*	ENSFALG00000000778	*NUCB2*
ENSFALG00000001676		ENSFALG00000000780	*PIK3C2A*
ENSFALG00000001682	*PRMT3*	ENSFALG00000000784	*RPS13*
ENSFALG00000001694	*SLC6A5*	ENSFALG00000000789	*PLEKHA7*
ENSFALG00000001705	*NELL1*	ENSFALG00000000793	
ENSFALG00000002080		ENSFALG00000000798	*SOX6*
ENSFALG00000002736	*ATP6V1G3*	ENSFALG00000001694	*SLC6A5*
ENSFALG00000002745	*NEK7*	ENSFALG00000001705	*NELL1*
ENSFALG00000002763	*LHX9*	ENSFALG00000001707	
ENSFALG00000002776		ENSFALG00000002080	
ENSFALG00000002788	*CRB1*	ENSFALG00000004750	*SLC17A6*
ENSFALG00000002807	*ZBTB41*	ENSFALG00000004784	*FANCF*
ENSFALG00000002825	*ASPM*	ENSFALG00000004792	*GAS2*
ENSFALG00000002859	*F13B*	ENSFALG00000005308	*PAPPA*
ENSFALG00000002866		ENSFALG00000006210	*WNT11*
ENSFALG00000002872	*KCNT2*	ENSFALG00000006210	*WNT11*
ENSFALG00000002879		ENSFALG00000006223	*THAP12*
ENSFALG00000003628	*YEATS2*	ENSFALG00000006228	*EMSY*
ENSFALG00000003638		ENSFALG00000006232	
ENSFALG00000003652		ENSFALG00000006235	*WDR73*
ENSFALG00000003657	*DUSP28*	ENSFALG00000006237	*ARRB1*
ENSFALG00000003666	*GPC1*	ENSFALG00000006241	*PDE2A*
ENSFALG00000003683		ENSFALG00000006247	*ARAP1*
ENSFALG00000004007		ENSFALG00000006250	*STARD10*
ENSFALG00000004008	*ADIPOQ*	ENSFALG00000006254	*CLPB*
ENSFALG00000004012	*ST6GAL1*	ENSFALG00000006255	*PHOX2A*
ENSFALG00000004018		ENSFALG00000006266	*INPPL1*
ENSFALG00000004578	*ZNF804A*	ENSFALG00000006276	
ENSFALG00000005821	*ADGRL3*	ENSFALG00000006281	
ENSFALG00000006299	*NUMA1*	ENSFALG00000006289	*LRTOMT*
ENSFALG00000006361	*GUCA1C*	ENSFALG00000006294	*LAMTOR1*
ENSFALG00000006362		ENSFALG00000006299	*NUMA1*
ENSFALG00000006365	*TRAT1*	ENSFALG00000006305	

ENSFALG00000006367	*SH2D1B*	ENSFALG00000006307	*RNF121*
ENSFALG00000006371	*KIAA1524*	ENSFALG00000006310	*C21orf62*
ENSFALG00000006405	*MYH15*	ENSFALG00000006314	*PAXBP1*
ENSFALG00000006425		ENSFALG00000006318	*BACH1*
ENSFALG00000006473		ENSFALG00000006320	
ENSFALG00000006474	*IFT57*	ENSFALG00000006323	*CCT8*
ENSFALG00000006475	*CD47*	ENSFALG00000006325	*USP16*
ENSFALG00000006478	*BBX*	ENSFALG00000006328	*LTN1*
ENSFALG00000007844	*CHST13*	ENSFALG00000006332	
ENSFALG00000007847	*TXNRD3*	ENSFALG00000006332	
ENSFALG00000007849	*PLXNA1*	ENSFALG00000006334	
ENSFALG00000007851	*CHCHD6*	ENSFALG00000006335	
ENSFALG00000007855	*TPRA1*	ENSFALG00000006337	
ENSFALG00000007857	*MCM2*	ENSFALG00000006339	*TMPRSS7*
ENSFALG00000008491	*IL17RD*	ENSFALG00000006341	*TAGLN3*
ENSFALG00000008510	*ARHGEF3*	ENSFALG00000006342	*ABHD10*
ENSFALG00000008523		ENSFALG00000006345	*PHLDB2*
ENSFALG00000008526	*CCDC66*	ENSFALG00000006349	*PLCXD2*
ENSFALG00000008530	*ERC2*	ENSFALG00000006353	
ENSFALG00000008532	*WNT5A*	ENSFALG00000006356	*NECTIN3*
ENSFALG00000008547	*CACNA2D3*	ENSFALG00000007605	
ENSFALG00000008568	*LRTM1*	ENSFALG00000007614	*MYOT*
ENSFALG00000011012	*NRXN1*	ENSFALG00000007626	*FAM13B*
ENSFALG00000011097	*ANO4*	ENSFALG00000007635	
ENSFALG00000011113	*ADAMTSL3*	ENSFALG00000007643	*WNT8A*
ENSFALG00000011122		ENSFALG00000007651	*NME5*
ENSFALG00000011124	*SLC5A8*	ENSFALG00000007654	*HNRNPAB*
ENSFALG00000011132	*SH3GL3*	ENSFALG00000007658	*PHYKPL*
ENSFALG00000011135	*UTP20*	ENSFALG00000007671	*COL23A1*
ENSFALG00000011147	*BNC1*	ENSFALG00000007682	*CLK4*
ENSFALG00000011156	*ARL1*	ENSFALG00000007687	*RASGEF1C*
ENSFALG00000011161		ENSFALG00000007690	*MAPK9*
ENSFALG00000011170	*NUP205*	ENSFALG00000007695	*GFPT2*
ENSFALG00000011182	*TM6SF1*	ENSFALG00000007699	*CNOT6*
ENSFALG00000011191	*BTBD1*	ENSFALG00000007706	*ADAMTS2*
ENSFALG00000011194	*CNOT4*	ENSFALG00000007724	*RUFY1*
ENSFALG00000011204		ENSFALG00000007746	*ASTN2*
ENSFALG00000011211	*FAM103A1*	ENSFALG00000007747	
ENSFALG00000011215	*HOMER2*	ENSFALG00000007747	
ENSFALG00000011224	*WHAMM*	ENSFALG00000007761	
ENSFALG00000011238		ENSFALG00000007764	
ENSFALG00000011252	*FSD2*	ENSFALG00000007769	*CANX*
ENSFALG00000011279	*STRA8*	ENSFALG00000007773	*MAML1*
ENSFALG00000011280	*PDE8A*	ENSFALG00000007777	
ENSFALG00000011292	*WDR91*	ENSFALG00000007786	*MGAT4B*
ENSFALG00000011312	*PDE6H*	ENSFALG00000007803	*SQSTM1*
ENSFALG00000011317	*ARHGDIB*	ENSFALG00000007812	*TBC1D9B*
ENSFALG00000011319	*GATM*	ENSFALG00000007816	*RNF130*
ENSFALG00000011328	*MGP*	ENSFALG00000007835	*FLT4*
ENSFALG00000011339	*HDC*	ENSFALG00000007835	*FLT4*

ENSFALG00000011345		ENSFALG00000007844	*CHST13*
ENSFALG00000011350	*ART4*	ENSFALG00000007847	*TXNRD3*
ENSFALG00000011355	*WBP11*	ENSFALG00000007849	*PLXNA1*
ENSFALG00000011359	*HIST1H4E*	ENSFALG00000007851	*CHCHD6*
ENSFALG00000011368		ENSFALG00000007855	*TPRA1*
ENSFALG00000011370	*GABPB1*	ENSFALG00000007857	*MCM2*
ENSFALG00000011372		ENSFALG00000007860	*PODXL2*
ENSFALG00000011378		ENSFALG00000007868	*ABTB1*
ENSFALG00000011384		ENSFALG00000007870	
ENSFALG00000011391	*DDX47*	ENSFALG00000007879	*HMGXB3*
ENSFALG00000011392	*USP8*	ENSFALG00000007883	*MGLL*
ENSFALG00000011403		ENSFALG00000007885	*KBTBD12*
ENSFALG00000011404	*USP50*	ENSFALG00000007889	*SEC61A1*
ENSFALG00000011415	*HEBP1*	ENSFALG00000007894	*RUVBL1*
ENSFALG00000011416	*TRPM7*	ENSFALG00000007901	*EEFSEC*
ENSFALG00000011427		ENSFALG00000007905	*CSF1R*
ENSFALG00000011434	*SPPL2A*	ENSFALG00000007910	*GATA2*
ENSFALG00000011447	*FAM234B*	ENSFALG00000007922	*RPN1*
ENSFALG00000011454	*CDC42SE1*	ENSFALG00000007930	*RAB7A*
ENSFALG00000011457		ENSFALG00000007933	
ENSFALG00000011462		ENSFALG00000007936	*HMCES*
ENSFALG00000011464	*GSG1*	ENSFALG00000007939	*PDGFRB*
ENSFALG00000011466	*EMP1*	ENSFALG00000007944	*CDX1*
ENSFALG00000011471	*BLOC1S6*	ENSFALG00000007951	*COPG1*
ENSFALG00000011480	*SLC30A4*	ENSFALG00000007961	*SLC6A7*
ENSFALG00000011485	*C15orf48*	ENSFALG00000007989	*CAMK2A*
ENSFALG00000011487	*SPATA5L1*	ENSFALG00000007992	*ARSI*
ENSFALG00000011497	*COPS2*	ENSFALG00000008004	
ENSFALG00000011503	*SECISBP2L*	ENSFALG00000008004	
ENSFALG00000011509	*SHC4*	ENSFALG00000008018	*CD74*
ENSFALG00000011512	*CEP152*	ENSFALG00000008029	*RPS14*
ENSFALG00000011520	*FBN1*	ENSFALG00000008032	*NDST1*
ENSFALG00000011538	*DUT*	ENSFALG00000008039	
ENSFALG00000011549	*SLC12A1*	ENSFALG00000008044	*MYOZ3*
ENSFALG00000011565	*MYEF2*	ENSFALG00000008051	*RBM22*
ENSFALG00000011584	*SLC24A5*	ENSFALG00000008066	*COL7A1*
ENSFALG00000011617	*SEMA6D*	ENSFALG00000008078	*DCTN4*
ENSFALG00000011894	*MYO5A*	ENSFALG00000008078	*DCTN4*
ENSFALG00000011902	*ARPP19*	ENSFALG00000008089	
ENSFALG00000011922	*FAM214A*	ENSFALG00000008095	*TNIP1*
ENSFALG00000011940	*ONECUT1*	ENSFALG00000008101	*SPOCK1*
ENSFALG00000011944	*WDR72*	ENSFALG00000008105	*KLHL3*
ENSFALG00000012394	*CBX7*	ENSFALG00000008225	*UQCRC1*
ENSFALG00000012402	*CBX6*	ENSFALG00000008229	*SLC26A6*
ENSFALG00000012405		ENSFALG00000008250	*CELSR3*
ENSFALG00000012410	*DNAL4*	ENSFALG00000008289	*NCKIPSD*
ENSFALG00000012424		ENSFALG00000008299	*IP6K2*
ENSFALG00000012447	*SUN2*	ENSFALG00000008310	*ABHD6*
ENSFALG00000012474	*GTPBP1*	ENSFALG00000008316	*DNASE1L3*
ENSFALG00000012479	*JOSD1*	ENSFALG00000008327	*FLNB*

ENSFALG00000012491	*TOMM22*	ENSFALG00000008337	*SLMAP*
ENSFALG00000012501	*CBY1*	ENSFALG00000008349	*DENND6A*
ENSFALG00000012518	*DMC1*	ENSFALG00000008385	*ARF4*
ENSFALG00000012560	*DDX17*	ENSFALG00000008401	*PDE12*
ENSFALG00000012578	*KDELR3*	ENSFALG00000008432	
ENSFALG00000012584	*KCNJ4*	ENSFALG00000008482	*ASB14*
ENSFALG00000012594		ENSFALG00000008483	*APPL1*
ENSFALG00000012601	*KLF15*	ENSFALG00000008487	*HESX1*
ENSFALG00000012603	*CFAP100*	ENSFALG00000008491	*IL17RD*
ENSFALG00000012608	*TMEM184B*	ENSFALG00000008510	*ARHGEF3*
ENSFALG00000012609		ENSFALG00000008510	*ARHGEF3*
ENSFALG00000012611	*MAFF*	ENSFALG00000008523	
ENSFALG00000012619	*UROC1*	ENSFALG00000008526	*CCDC66*
ENSFALG00000012626		ENSFALG00000008530	*ERC2*
ENSFALG00000012631	*CHCHD4*	ENSFALG00000008532	*WNT5A*
ENSFALG00000012632	*PLA2G6*	ENSFALG00000008547	*CACNA2D3*
ENSFALG00000012635		ENSFALG00000008568	*LRTM1*
ENSFALG00000012642	*XPC*	ENSFALG00000009947	
ENSFALG00000012644	*LSM3*	ENSFALG00000009951	*LRRC38*
ENSFALG00000012649	*SLC6A6*	ENSFALG00000009952	
ENSFALG00000012656	*GRIP2*	ENSFALG00000009955	
ENSFALG00000012664	*BAIAP2L2*	ENSFALG00000009960	
ENSFALG00000012685	*SLC16A8*	ENSFALG00000009964	
ENSFALG00000012698	*PICK1*	ENSFALG00000009969	*DHRS3*
ENSFALG00000012706	*SOX10*	ENSFALG00000009972	*VPS13D*
ENSFALG00000012726	*MICALL1*	ENSFALG00000009989	
ENSFALG00000012747	*C22orf23*	ENSFALG00000009999	
ENSFALG00000012758		ENSFALG00000010006	*MFN2*
ENSFALG00000012768	*EIF3L*	ENSFALG00000010022	*PLOD1*
ENSFALG00000012782	*ANKRD54*	ENSFALG00000010046	*KIAA2013*
ENSFALG00000012788	*GALR3*	ENSFALG00000010060	
ENSFALG00000012790	*GCAT*	ENSFALG00000010072	
ENSFALG00000012797		ENSFALG00000010074	
ENSFALG00000012807		ENSFALG00000010079	
ENSFALG00000012816		ENSFALG00000010087	*CLCN6*
ENSFALG00000012823		ENSFALG00000010091	*MTHFR*
ENSFALG00000012836	*SH3BP1*	ENSFALG00000010093	*AGTRAP*
ENSFALG00000012856	*GGA1*	ENSFALG00000010100	*DRAXIN*
ENSFALG00000012864	*CDC42EP1*	ENSFALG00000010104	*MAD2L2*
ENSFALG00000012867	*LGALS2*	ENSFALG00000010110	
ENSFALG00000012877	*CARD10*	ENSFALG00000010113	*FBXO2*
ENSFALG00000012883	*MFNG*	ENSFALG00000010115	*DISP3*
ENSFALG00000012885	*CYTH4*	ENSFALG00000010131	*UBIAD1*
ENSFALG00000012894	*RAC2*	ENSFALG00000010131	*UBIAD1*
ENSFALG00000012908	*C1QTNF6*	ENSFALG00000010184	*MTOR*
ENSFALG00000012918	*IL2RB*	ENSFALG00000010279	*ANGPTL7*
ENSFALG00000012926	*TMPRSS6*	ENSFALG00000010287	*EXOSC10*
ENSFALG00000012941	*KCTD17*	ENSFALG00000010293	*SRM*
ENSFALG00000012951	*MPST*	ENSFALG00000010295	*PPIH*
ENSFALG00000012956	*TST*	ENSFALG00000010305	*YBX1*

ENSFALG00000012959		ENSFALG00000010305	*YBX1*
ENSFALG00000012962		ENSFALG00000010324	
ENSFALG00000012982	*NCF4*	ENSFALG00000010324	
ENSFALG00000013256	*COL15A1*	ENSFALG00000010331	*CLDN19*
ENSFALG00000013285	*TGFBR1*	ENSFALG00000010338	*P3H1*
ENSFALG00000013290		ENSFALG00000010347	*C1orf50*
ENSFALG00000013292		ENSFALG00000010353	*CDC42*
ENSFALG00000013297		ENSFALG00000010357	*WNT4*
ENSFALG00000013299	*ADNP2*	ENSFALG00000011059	*SERGEF*
ENSFALG00000013303	*RBFA*	ENSFALG00000011078	*TPH1*
ENSFALG00000013312		ENSFALG00000011111	*SAAL1*
ENSFALG00000013317		ENSFALG00000011119	
ENSFALG00000013319		ENSFALG00000011125	*HPS5*
ENSFALG00000013321	*TXNL4A*	ENSFALG00000011148	*GTF2H1*
ENSFALG00000013323	*HSBP1L1*	ENSFALG00000011161	
ENSFALG00000013325	*PQLC1*	ENSFALG00000011162	
ENSFALG00000013326	*KCNG2*	ENSFALG00000011179	*TSG101*
ENSFALG00000013330	*CTDP1*	ENSFALG00000011182	*TM6SF1*
ENSFALG00000013331	*ITGB2*	ENSFALG00000011191	*BTBD1*
ENSFALG00000013336	*FAM207A*	ENSFALG00000011204	
ENSFALG00000013340	*ADARB1*	ENSFALG00000011211	*FAM103A1*
ENSFALG00000013410	*CHERP*	ENSFALG00000011215	*HOMER2*
ENSFALG00000013412		ENSFALG00000011218	*UEVLD*
ENSFALG00000013413	*EPS15L1*	ENSFALG00000011224	*WHAMM*
ENSFALG00000014557	*CTXN2*	ENSFALG00000011252	*FSD2*
ENSFALG00000014633		ENSFALG00000011262	*SPTY2D1*
ENSFALG00000014691		ENSFALG00000011276	*TMEM86A*
ENSFALG00000014830		ENSFALG00000011280	*PDE8A*
ENSFALG00000014846		ENSFALG00000011286	*PTPN5*
ENSFALG00000014856	*SMCO3*	ENSFALG00000011297	*PTPRJ*
ENSFALG00000014871		ENSFALG00000011319	*GATM*
ENSFALG00000014894		ENSFALG00000011339	*HDC*
ENSFALG00000014910	*HIST1H4G*	ENSFALG00000011370	*GABPB1*
ENSFALG00000014919		ENSFALG00000011392	*USP8*
ENSFALG00000014939		ENSFALG00000011404	*USP50*
ENSFALG00000014961		ENSFALG00000011416	*TRPM7*
ENSFALG00000014977		ENSFALG00000011468	*GRIN2B*
ENSFALG00000015009		ENSFALG00000011483	*ATF7IP*
ENSFALG00000015030		ENSFALG00000011495	*PLBD1*
ENSFALG00000015045		ENSFALG00000011520	*FBN1*
ENSFALG00000015063		ENSFALG00000011538	*DUT*
ENSFALG00000015083		ENSFALG00000011538	*DUT*
ENSFALG00000015094		ENSFALG00000011542	*GUCY2C*
ENSFALG00000015107		ENSFALG00000011542	*GUCY2C*
ENSFALG00000015108		ENSFALG00000011549	*SLC12A1*
ENSFALG00000015121		ENSFALG00000011565	*MYEF2*
ENSFALG00000015142	*APOLD1*	ENSFALG00000011584	*SLC24A5*
ENSFALG00000015202	*H1F0*	ENSFALG00000011617	*SEMA6D*
ENSFALG00000015212	*ELFN2*	ENSFALG00000011669	*DMXL2*
ENSFALG00000015222	*SSTR3*	ENSFALG00000011679	*GLDN*

ENSFALG00000011696	*CYP19A1*
ENSFALG00000011728	*TNFAIP8L3*
ENSFALG00000011736	*AP4E1*
ENSFALG00000011743	*SCG3*
ENSFALG00000011748	*LYSMD2*
ENSFALG00000011763	*TMOD2*
ENSFALG00000011807	*TMOD3*
ENSFALG00000011844	*LEO1*
ENSFALG00000011855	*MAPK6*
ENSFALG00000011866	*GNB5*
ENSFALG00000011882	*MYO5C*
ENSFALG00000011894	*MYO5A*
ENSFALG00000011902	*ARPP19*
ENSFALG00000011910	*UVRAG*
ENSFALG00000011922	*FAM214A*
ENSFALG00000011922	*FAM214A*
ENSFALG00000011940	*ONECUT1*
ENSFALG00000012601	*KLF15*
ENSFALG00000012603	*CFAP100*
ENSFALG00000012609	
ENSFALG00000012619	*UROC1*
ENSFALG00000012626	
ENSFALG00000012631	*CHCHD4*
ENSFALG00000012635	
ENSFALG00000012642	*XPC*
ENSFALG00000012644	*LSM3*
ENSFALG00000012649	*SLC6A6*
ENSFALG00000012656	*GRIP2*
ENSFALG00000012673	*CCDC174*
ENSFALG00000012692	
ENSFALG00000012697	*CCDC51*
ENSFALG00000012708	*PLXNB1*
ENSFALG00000014233	
ENSFALG00000014234	
ENSFALG00000014359	
ENSFALG00000014362	
ENSFALG00000014366	
ENSFALG00000014369	*SMIM3*
ENSFALG00000014395	*HNRNPA0*
ENSFALG00000014482	
ENSFALG00000014557	*CTXN2*
ENSFALG00000014588	
ENSFALG00000014605	*KCNJ11*
ENSFALG00000014633	
ENSFALG00000014651	*DNAJB8*
ENSFALG00000014671	*H1FX*
ENSFALG00000014697	*HIST2H2AB*
ENSFALG00000014713	
ENSFALG00000014787	

Supplementary Table 6B.

Summary of the gene ontology enrichment analysis for genes located in the relative differentiation (F_{ST}^{Z}) outliers between allopatric and sympatric *abietinus* and *tristis*. The orthologous *Ficedula albicollis* ensemble entry ID:s and the corresponding gene name is given (when available). P-value is corrected for multiple testing using the Benjamini and Hochberg (1995) method and the enrichment score is calculated as (o/O)/(e/E) where o = number of genes associated with term in differentiation outlier regions, O = total number of genes in differentiation outlier regions, e = number of genes associated with the term in the entire gene, and, E = total number of genes in the entire gene set.

**Ontology term**	**Genes**	**P-value**	**Enrichment**
GO:0051128<br>Biological process<br>*Regulation of cellular component organization*	*WNT11, ARRB1, ARAP1, INPPL1, PHLDB2, NOVEL, NOVEL, MAD2L2, MTOR, CDC42, WNT4, WHAMM*	0.00379	5.19
GO:0051128<br>Biological process<br>*Regulation of cellular component organization*	*WNT11, ARRB1, ARAP1, PAXBP1, CCT8, PHLDB2, GATA2, NOVEL, MTHFR, MTOR, EXOSC10, CDC42, WNT4, NOVEL, WHAMM, TMOD2, TMOD3, PLXNB1*	0.00379	2.89

Supplementary Table 7.

Alphabetically ordered list of genes containing fixed differences at non-synonymous (n = 18 sites) and splice regulating sites (n = 17) between allopatric *abietinus* and *tristis* as identified using SnpEff version 4.3 (Cingolani *et al.* 2012). Gene function is according to the biological process / molecular function in Ensembl (http://www.ensembl.org/index.html). # indicates the number of sites in each specific gene.

Gene	EnsemblID	#	Gene function
NON-SYN			
ATF7IP	ENSFALG00000011483	1	DNA methylation, negative regulation
DAPK1	ENSFALG00000003534	1	Signal transduction
DENND4C	ENSFALG00000003075	1	Nucleotide exchange factor
EXOSC10	ENSFALG00000010287	1	RNA processing
FYB	ENSFALG00000002027	2	Immune response
KDM4C	ENSFALG00000010643	1	Regulation of expression / Histone methylation
KIF24	ENSFALG00000009881	1	Intracellular transport / Microtubule binding
MACF1	ENSFALG00000000559	1	Cytoskeletal bridges
OSMR	ENSFALG00000002039	1	Regulation of cell proliferation
PIK3C3	ENSFALG00000008849	1	Transferase activity and binding
PRUNE2	ENSFALG00000003236	2	Pyrophosphatase activity / lipid metabolism
SLC27A3	ENSFALG00000003199	1	Fatty acid metabolism
TCF20	ENSFALG00000011651	1	Transcription factor / RNA binding
TPH1	ENSFALG00000011078	1	Aromatic amino acid metabolism
TXN	ENSFALG00000000191	1	Glycerol metabolism
ZCCHC6	ENSFALG00000003541	1	RNA processing
SPLICE			
ABCA1	ENSFALG00000004938	1	Protein lipidation / Cholesterol metabolism

C5orf42	ENSFALG00000002055	1	Unknown
C9orf84	ENSFALG00000000166	1	Unknown
CAP1	ENSFALG00000000523	1	Actin binding
CEP85	ENSFALG00000000387	1	Regulation of kinase activity
FANCC	ENSFALG00000003492	1	DNA repair
HOOK3	ENSFALG00000005146	1	Intracellular transport / Microtubule binding
KDELR3	ENSFALG00000012578	1	Endoplasmic reticulum receptor
KDM4C	ENSFALG00000010643	1	Regulation of expression / Histone methylation
KIF24	ENSFALG00000009881	1	Intracellular transport / Microtubule binding
NAA35	ENSFALG00000003574	1	Regulation of apoptosis / Muscle cell activity
OSTF1	ENSFALG00000012650	1	Bone resorption
PLOD1	ENSFALG00000010022	1	Collagen formation
SECISBP2	ENSFALG00000012441	1	RNA binding / neuron development
SLC1A1	ENSFALG00000010522	1	Transmembrane transport
TRNAU1AP	ENSFALG00000001371	1	Nucleic acid binding / Selenocysteine incorporation
XPNPEP3	ENSFALG00000012055	1	Aminopeptidase activity / Protein processing

Supplementary Figure 1.

Comparison of the annotation of the chiffchaff and the *Ficedula albicollis* mtDNA genome using the web-based tool MITOS (Bernt *et al.* 2013). a) Positions of the coding genes along the mtDNA assembly, and, b) positions of non-coding (rRNA and tRNA) genes in the mtDNA assemblies of chiffchaff (top) and collared flycatcher (bottom), respectively.

Chiffchaff (*Phylloscopus collybita abietinus*)
mtDNA genome assembly coding gene positions

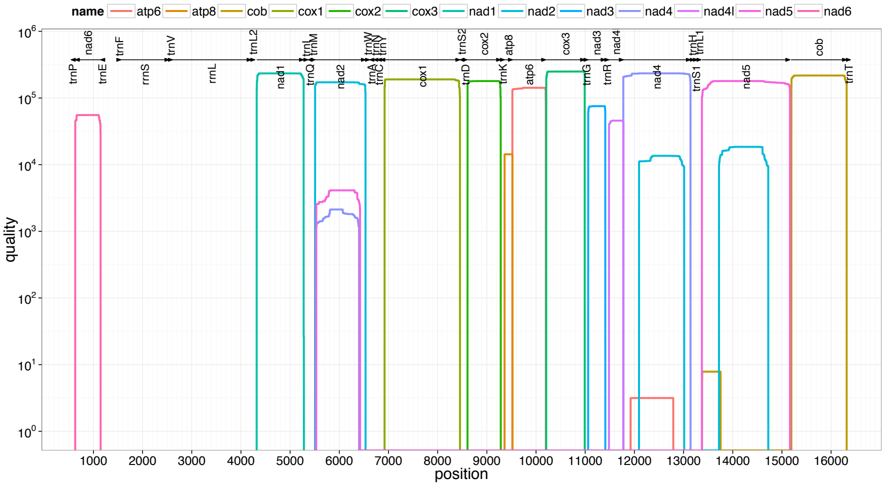

Collared flycatcher (*Ficedula albicollis*)
mtDNA genome assembly coding gene positions

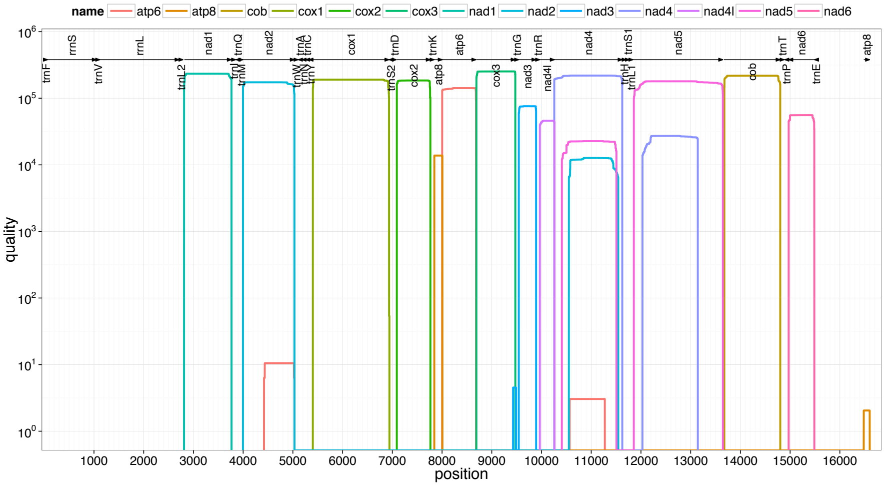

a)

Chiffchaff (*Phylloscopus collybita abietinus*)
mtDNA non-coding (RNA) gene positions

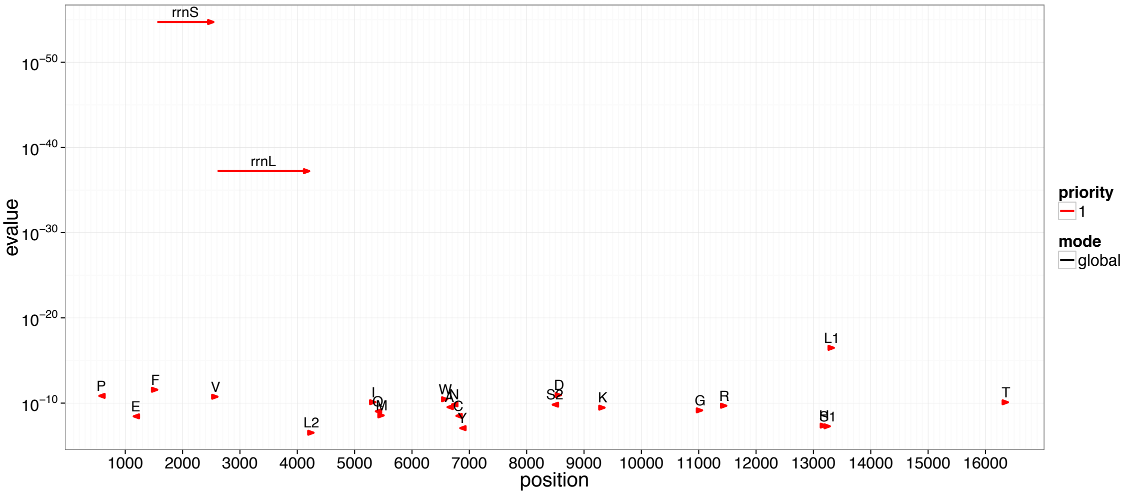

Collared flycatcher (*Ficedula albicollis*)
mtDNA non-coding (RNA) gene positions

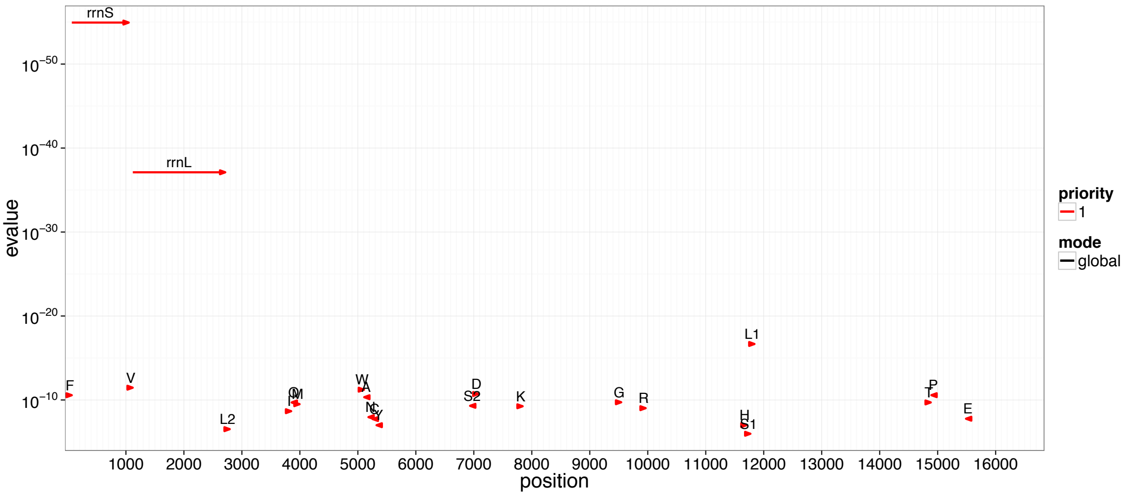

b)

Supplementary Figure 2.

A phylogeny based on the mtDNA whole genome alignments with information about sampling location for all 40 individuals included in the study. Yellow dots = allopatric *abietinus*, blue dots = sympatric *abietinus*, brown dots = allopatric *tristis* and green dots = sympatric *tristis*. Sampling sites for individuals are indicated to the right in the graph with allopatric *abietinus* and *tristis* in brown and yellow font, respectively and individuals from the Northern (N) and Southern (S) sympatric zone in blue and green font, respectively. The scale bar at the bottom indicates the number of substitutions per site.

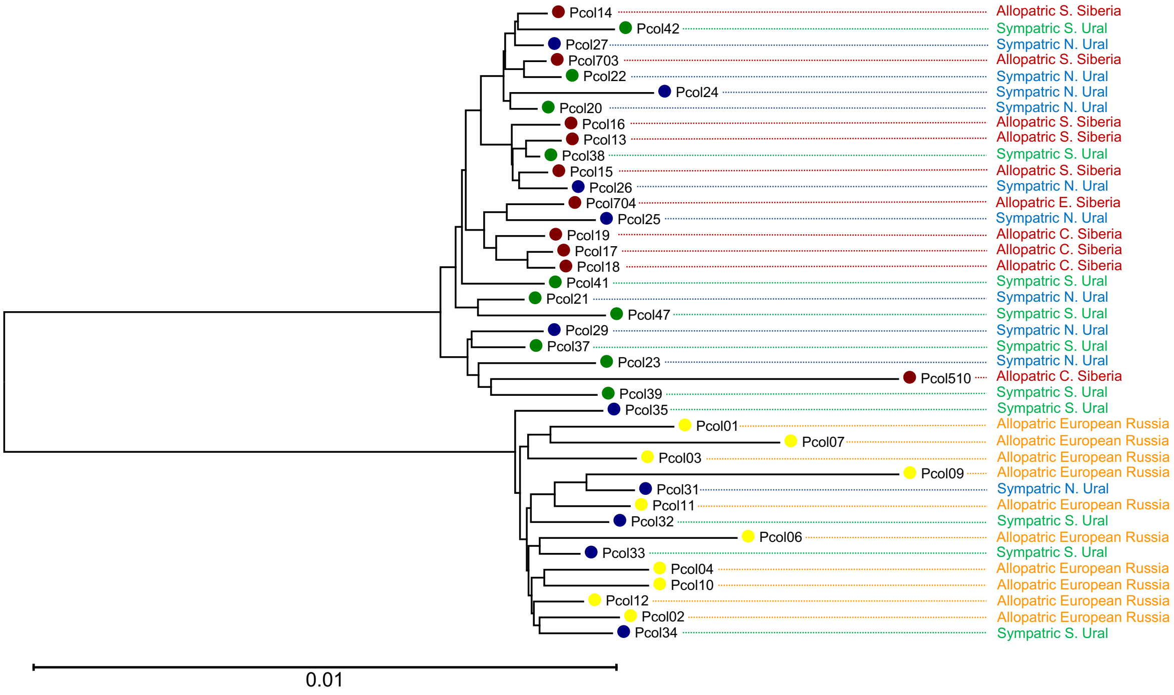

Supplementary Figure 3.

Scatter plots showing the decay in linkage disequilibrium (LD) in each respective population: allopatric *abietinus* (yellow, top left), allopatric *tristis* (brown, top right), sympatric *abietinus* (blue, bottom left), and sympatric *tristis* (green, bottom right). LD is estimated as the correlation between alleles (r^2) across SNPs < 100 kb apart and with a minor allele frequency > 0.20.

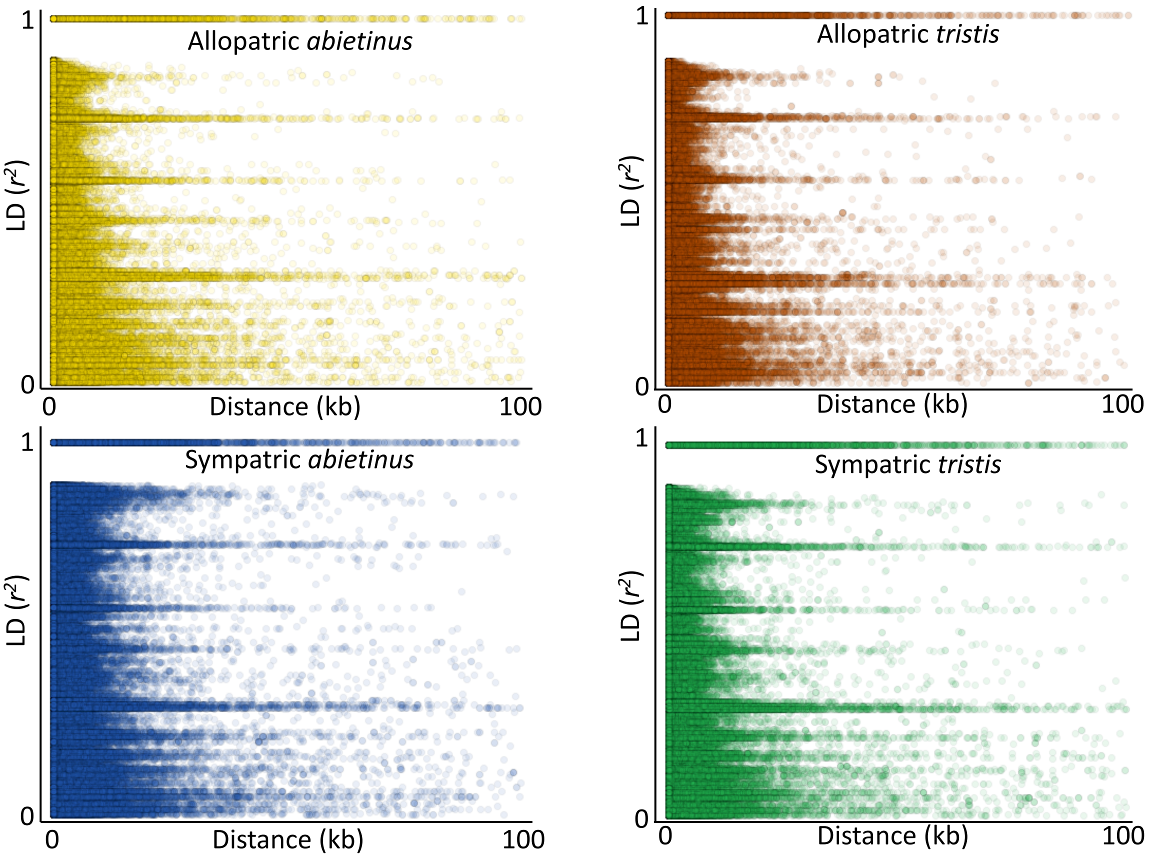

Supplementary Figure 4.

An illustration of the genome scan between allopatric and sympatric populations of the *abietinus* and *tristis* species pair. Each segment represents one chromosome and estimates of the number of fixed differences (# *Fix*, allopatry only, top panel) genetic differentiation (F_{ST}, second panel), absolute divergence (D_{XY}, middle panel), genetic diversity (Θ_{π}, fourth panel) and Tajima's *D* (*Taj D*, bottom panel), estimated in 10 kb windows across the chromosomes, are presented. Allopatric comparisons are indicated with gray lines and sympatric comparisons with red lines (F_{ST} and D_{XY}) and the intra-population summary statistics (Θ_{π} and *TajD*) are colored as follows; allopatric *abietinus* = yellow, allopatric *tristis* = brown, sympatric *abietinus* = blue, sympatric *tristis* = green.

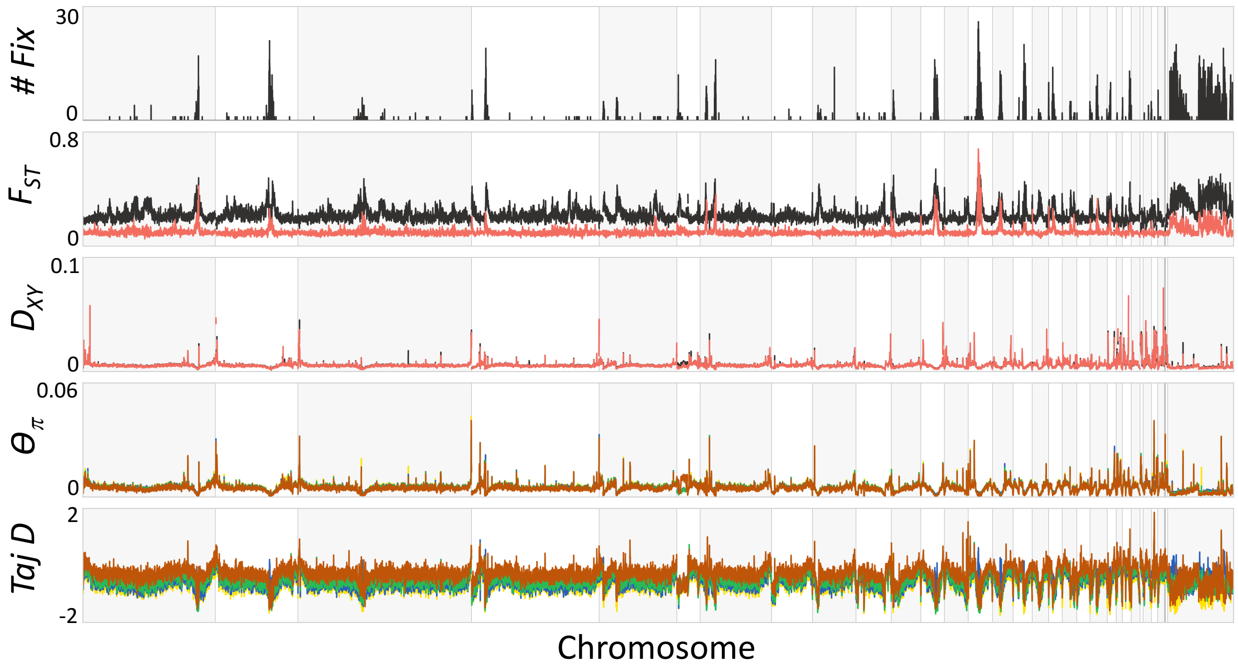

Supplementary Figure 5.

Correlation between genetic differentiation (F_{ST}) and absolute divergence (D_{XY}) in the comparison of allopatric (gray dots, top plot, Pearson's r = -0.225, p-value < $2.2*10^{-16}$) and sympatric (red dots, bottom plot, Pearson's r = -0.130, p-value < $2.2*10^{-16}$) *abietinus* and *tristis*.

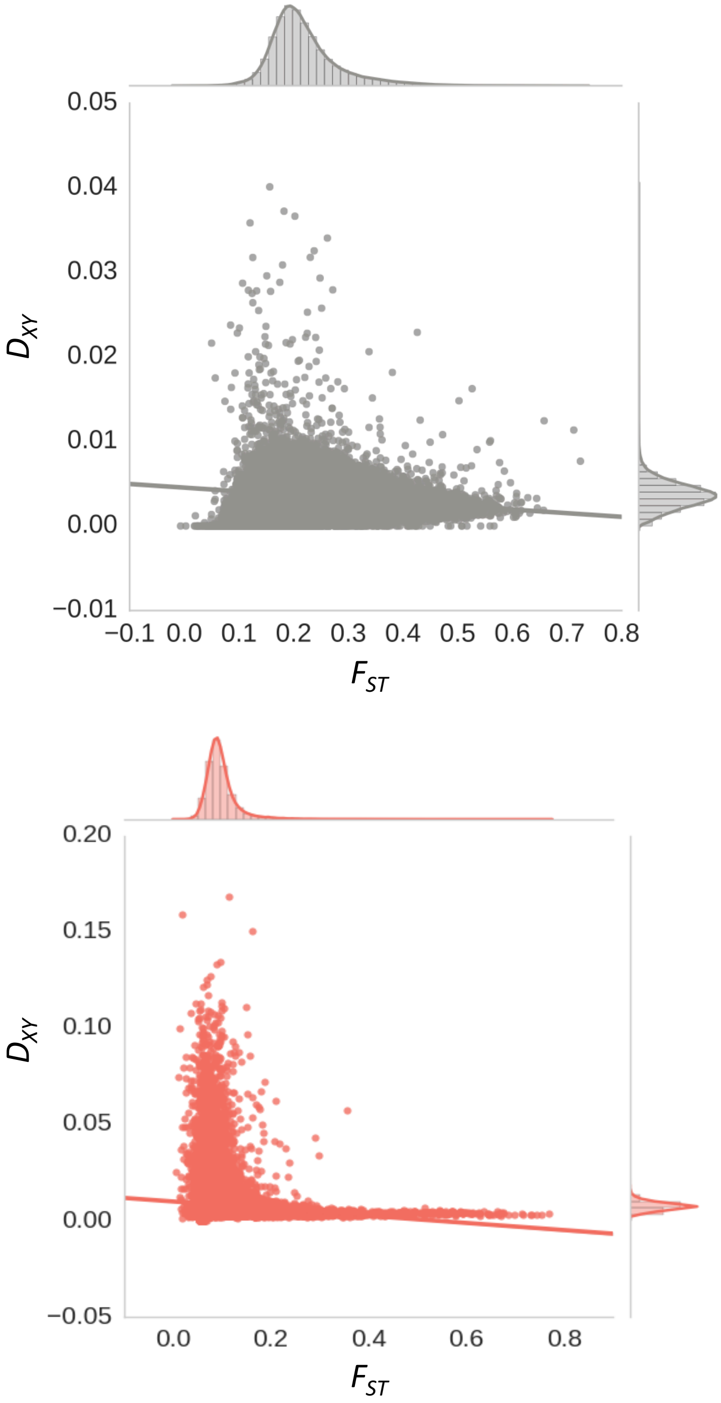

Supplementary Figure 6.

Correlation between population specific Tajima's D (Y-axis) and genetic differentiation (F_{ST}, x-axis) in the allopatric (top panels, gray) and sympatric (bottom panels, red) comparisons. Details of the correlations are as follows:

Allopatric *abietinus*: Pearson's r = -0.070, t = -22.604, df = 103,770, p-value < $2.2*10^{-16}$

Allopatric *tristis*: Pearson's r = -0.166, t = -54.245, df = 103,770, p-value < $2.2*10^{-16}$

Sympatric *abietinus*: Pearson's r = 0.162, t = 53.076, df = 103,710, p-value < $2.2*10^{-16}$

Sympatric *tristis*: Pearson's r = -0.166, t = -54.047, df = 103,710, p-value < $2.2*10^{-16}$

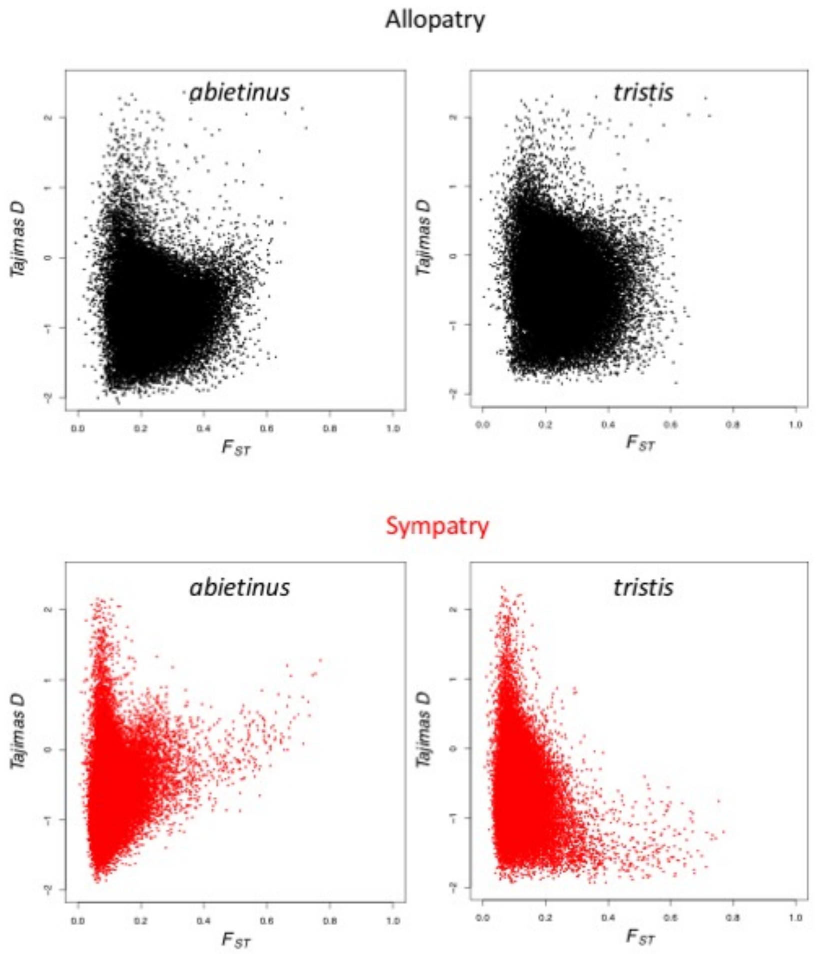

Supplementary Figure 7.

Illustration of the correlation between average genetic diversity (Θ_π) and absolute divergence (D_{XY}) in the allopatric (top panels, gray) and sympatric (bottom panels, red) comparisons for the autosomes (left column) and the Z-chromosome (right column), respectively. The solid line in each plot indicates the 1:1 relationship. The level of genetic differentiation (F_{ST}) is indicated by the intensity of the color on the dots in each plot from low differentiation (pale gray or pale red) to high differentiation (dark gray or dark red).

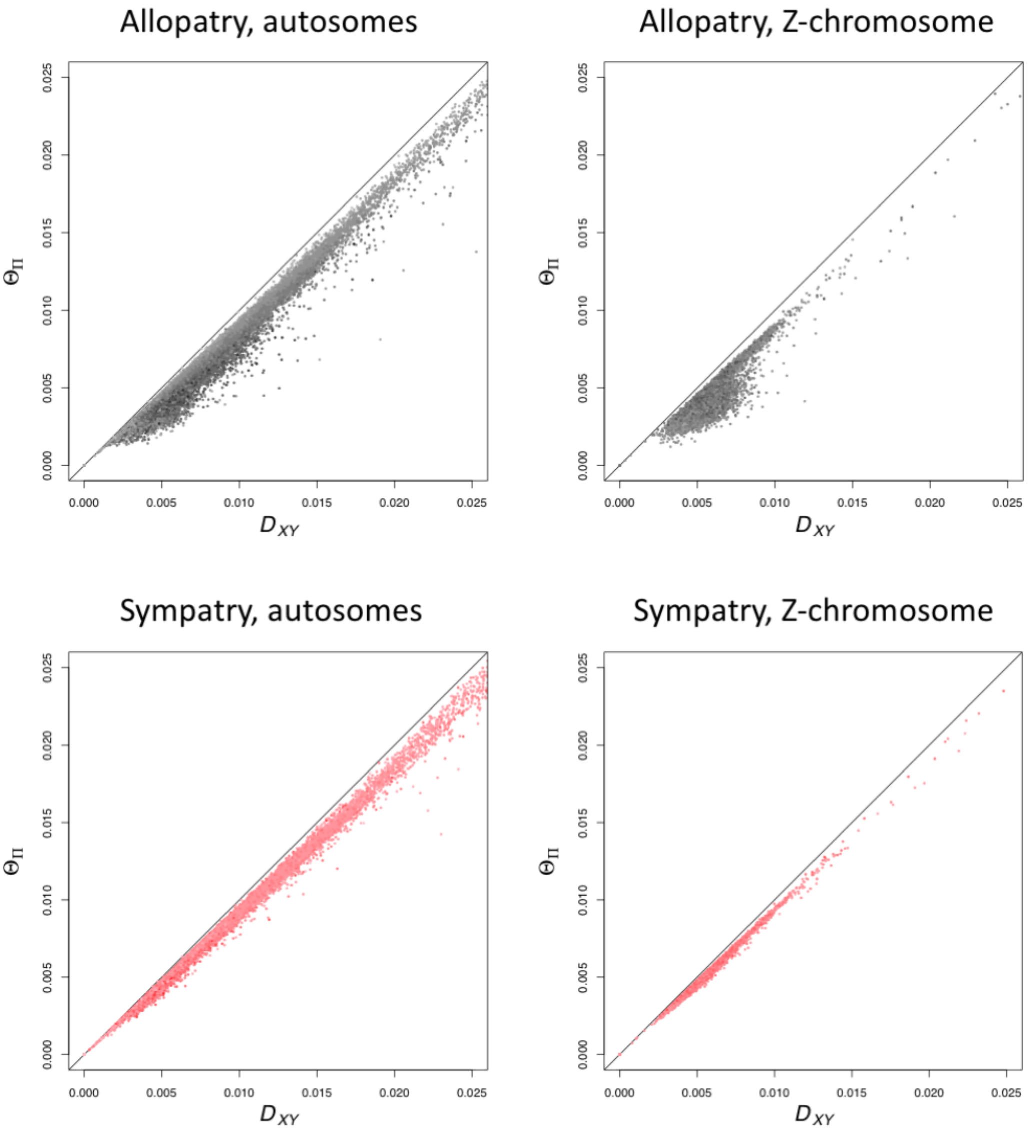

Supplementary Figure 8.

Detailed illustration of five potential structural rearrangements between *F. albicollis* and *P. abietinus* involving chromosomes 3, 4, 5, 20 and Z. The sudden changes (dotted vertical lines) in levels of genetic variation likely reflect break-points of inversions.

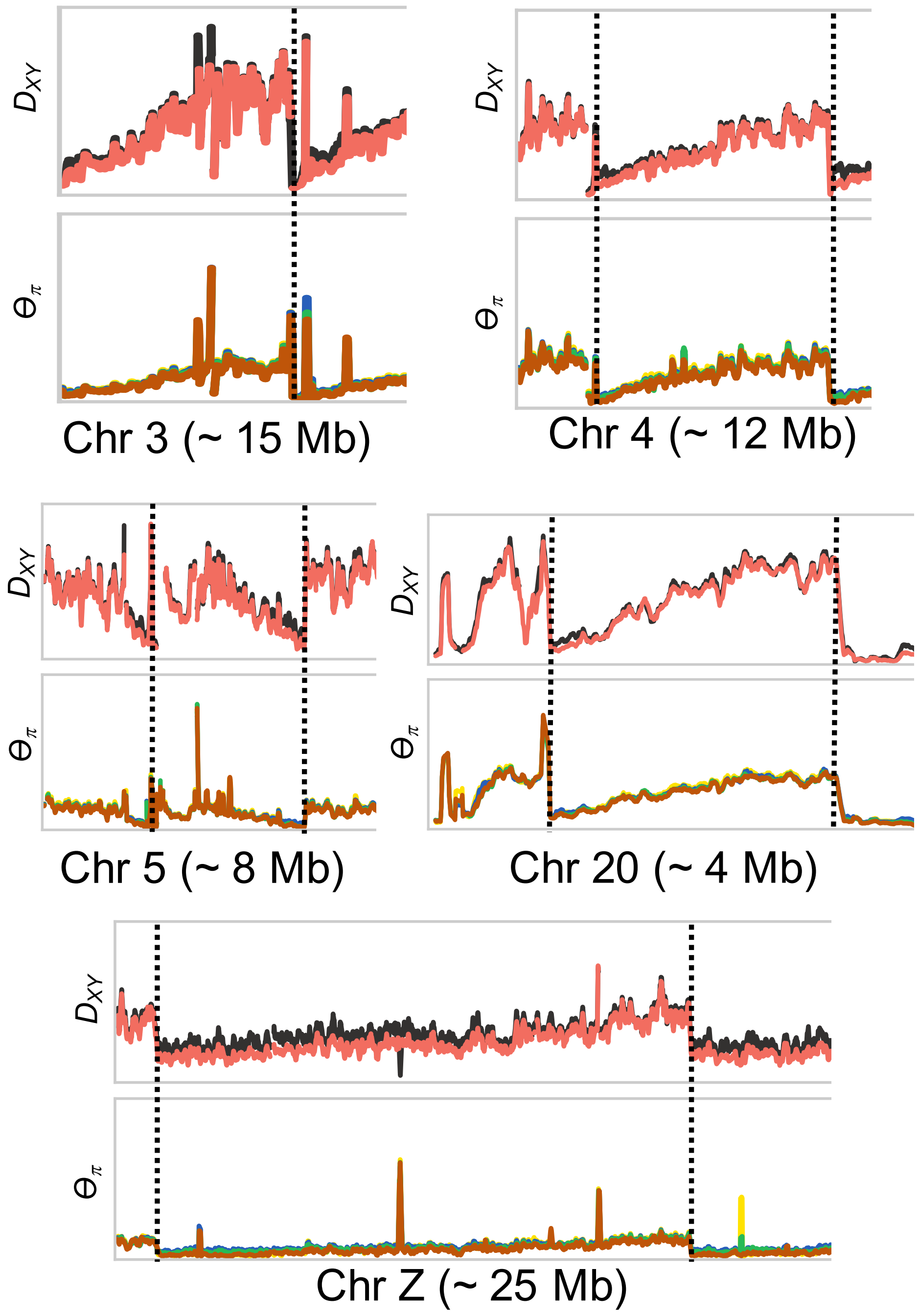

Supplement: Supplementary file 1 [file 3983FileS1.pdf]
